# Developmental Origins of Human Cortical Oligodendrocytes and Astrocytes

**DOI:** 10.1007/s12264-021-00759-9

**Published:** 2021-08-10

**Authors:** Lin Yang, Zhenmeiyu Li, Guoping Liu, Xiaosu Li, Zhengang Yang

**Affiliations:** State Key Laboratory of Medical Neurobiology and MOE Frontiers Center for Brain Science, Institutes of Brain Science, Institute for Translational Brain Research, and Department of Neurology, Zhongshan Hospital, Fudan University, Shanghai, 200032 China

**Keywords:** Radial glial cell, Intermediate progenitor cell, EGFR, HOPX, Oligodendrocyte, Astrocyte, Olfactory bulb interneuron, Cerebral cortex, Human

## Abstract

Human cortical radial glial cells are primary neural stem cells that give rise to cortical glutaminergic projection pyramidal neurons, glial cells (oligodendrocytes and astrocytes) and olfactory bulb GABAergic interneurons. One of prominent features of the human cortex is enriched with glial cells, but there are major gaps in understanding how these glial cells are generated. Herein, by integrating analysis of published human cortical single-cell RNA-Seq datasets with our immunohistochemistical analyses, we show that around gestational week 18, EGFR-expressing human cortical truncated radial glial cells (tRGs) give rise to basal multipotent intermediate progenitors (bMIPCs) that express EGFR, ASCL1, OLIG2 and OLIG1. These bMIPCs undergo several rounds of mitosis and generate cortical oligodendrocytes, astrocytes and olfactory bulb interneurons. We also characterized molecular features of the cortical tRG. Integration of our findings suggests a general picture of the lineage progression of cortical radial glial cells, a fundamental process of the developing human cerebral cortex.

**Supplementary Information:**

The online version contains supplementary material available at 10.1007/s12264-021-00759-9.

## Introduction

Radial glial cells (RGCs) are the primary stem cells during neural development [1–3]. They have a bipolar shape with their shorter apical process contacting with the ventricular surface and the much longer basal (outer) process contacting with the pial surface [4, 5]. During mammalian cortical development, cortical RGCs sequentially generate the laminar subtypes of glutaminergic projection pyramidal neurons (PyNs), cortical oligodendrocytes, astrocytes, and olfactory bulb interneurons (OBiNs) [2, 6–9].

The lineage progression of the cortical RGC in the mouse brain has now defined. Cortical (pallial) neuroepithelial cells transform into cortical RGCs, which undergo asymmetric cell divisions to self-renew and to produce short neural precursors (SNPs) around E11.5-E16.5 [10–12]. SNPs, a transitional cell form between cortical RGCs and intermediate progenitor cells (IPCs), are apical IPCs as they possess a ventricular endfoot and a basal process of variable length [10–15]. SNPs give rise to PyN-IPCs, which are unipotent and exclusively generate PyNs [7, 16–19]. Later in prenatal development, the properties of mouse cortical RGCs undergo a major switch and start to produce a different type of IPC that is multipotent. They generate cortical oligodendrocytes, astrocytes and OBiNs, which we have termed multipotent IPCs (MIPCs) [6]. Around embryonic day (E)16.5, mouse cortical RGCs give rise to EGFR^+^ASCL1^+^ apical MIPCs (aMIPCs) in the ventricular zone (VZ) and subventricular zone (SVZ). aMIPCs, are transitional cells which then differentiate into basal MIPCs (bMIPCs) that coexpress EGFR, ASCL1, OLIG2 and OLIG1. bMIPCs undergo several rounds of mitosis and generate cortical oligodendrocyte-lineage-restricted IPCs (OPCs), astrocyte lineage-restricted IPCs (APCs), and OBiN lineage-restricted IPCs (OBiN-IPCs). These lineage-restricted IPCs then divide symmetrically to generate cortical astrocytes, oligodendrocytes and OBiNs, respectively [6, 20].

During human cortical development, three types of RGCs have been identified. At the onset on PyN genesis around gestational week 7 (GW7), human cortical neuroepithelial cells transform into the ventricular radial glial cells (vRGs, also referred to as apical RGs). Like mouse cortical RGCs, human cortical vRGs have apical-basal polarity, contacting both the ventricle and the pia [4, 21–26]. Around GW15-GW16, vRGs begin to generate outer radial glia cells (oRGs) and truncated radial glial cells (tRGs); as a result the radial glia scaffold becomes discontinuous [27, 28]. oRGs (also known as basal RGs) no longer contact the ventricle but they directly inherit the basal process from their vRG mother cells, thereby maintaining contact the pial surface. On the other hand, tRGs no longer contact the pia, and their basal processes mainly terminate in the outer SVZ (OSVZ) [27]; they inherit the apical domain from vRGs, which contact the lateral ventricle throughout the development. vRGs mainly generate deep layer PyNs, whereas oRGs mainly generate upper layer PyNs [21, 26, 27, 29, 30]. The progeny of tRGs is unknown.

A prominent feature of human brain is its relative enrichment with macroglial cells (oligodendrocytes and astrocytes), which make up at least 50% of the cells in the human cortex (approximately 20% more than in mouse cortex) [31–34]. However, the developmental origins of cortical glial cells and cortex-derived OBiNs in the human brain remain largely unknown. Here we explore whether recent findings from the mouse [6] shed light on this subject. We re-analyzed published single-cell RNA-Seq datasets, including 3,355 EGFR^+^ cells from human frontal cortex at GW21-GW26 [35] and 33,976 cells from human cortex at GW17-GW18 [36], in the context of our immunohistochemistry results, and provide evidence that human cortical EGFR^+^ tRGs generate bMIPCs that coexpress EGFR, ASCL1, OLIG2 and OLIG1. These MIPCs in turn give rise to cortical oligodendrocytes, astrocytes and OBiNs. Thus, we conclude that developmental origins of human cortical glial cells are similar to that in the mouse cortex. Further, we present a general model of RGC lineage progression and molecular identity of tRGs in the human cerebral cortex.

## Materials and Methods

### Human Tissue Collection

Three fetal brain samples (GW18, *n* = 1, male; GW23, *n* = 2, male and female) were collected with informed consent and in accordance with the Fudan University Shanghai Medical College guidelines, and the study design was approved by the institutional review board (ethics committee) of the Fudan University Shanghai Medical College (20110307-085 and 20120302-099). Fetal brains were obtained at autopsy within 3–5 h of spontaneous abortion, fixed in 4% PFA at 4°C for 3–7 days and then cryoprotected in 30% sucrose in 0.1 mol/L phosphate buffer for 72 h at 4°C. The brain tissue samples were frozen in in embedding medium (OCT; Sakura Finetek) on a dry ice and ethanol slush. All of these 3 fetal brain samples had been used in previous study [37, 38].

### Immunohistochemistry

Immunohistochemistry was performed using standard protocols [6, 37]. Immunohistochemical staining was performed on 60 μm thick fetal brain coronal sections which were mounted on glass slides. E17.5 and E18.5 mouse brains were cryosectioned into thickness of 12 μm. In the present study, all human brain sections were subjected to an antigen retrieval protocol (slides were briefly boiled in 10 mmol/L sodium citrate, pH 6.0). Sections were then permeabilized with 0.05% Triton X-100 for 1 h, followed by an incubation in blocking buffer (5% donkey serum and 0.05% Triton X-100 in TBS) for 2 h. The blocking buffer was removed, and the sections were incubated with primary antibodies (diluted in the blocking buffer) for 24–48 h at 4°C. The sections were washed in TBS, and incubated with secondary antibodies conjugated to Alexa Fluor488, Cy3 or Cy5 for 2 h at room temperature. All secondary antibodies were from Jackson ImmunoResearch. Finally, the sections were counterstained with DAPI for 2 mins before being mounted in the fluorescence mounting medium (DAKO S3023).

The following primary antibodies were used in this study: rabbit anti-ASCL1 (1:1,000, Cosmo Bio, SKT01-003), rabbit anti-OLIG2 (1:500, Millipore, AB9610), mouse anti-OLIG2 (1:500, Millipore MABN50), goat anti-EGFR (1:500, R&D System, BAF1280, for mouse brain sections), goat anti-EGFR (1:1,000, R&D System, AF231, for human brain sections), sheep anti EOMES (1:1,000, R&D System, AF6166); rabbit anti EOMES (1:1,000, Abcam, ab23345); rat anti EOMES (1:300, Thermo Fisher, 12-4875-82, only for mouse brain sections), mouse anti PAX6 (1:500, BD Biosciences, 561664), mouse anti CRYAB (1:300, Abcam, ab13496); goat anti SOX10 (1:500, R&D System, AF2864), chicken anti GFAP (1:1,500, Abcam, ab4674), rabbit anti HOPX (1:300, Proteintech, 11419-1-AP), rabbit anti GSX2 (1:1,000, Millipore, ABN162), rabbit anti GABA (1:1,000, Sigma-Aldrich, A2052), goat anti SP8 (1:2,000, Santa Cruz, sc-104661), goat anti PDGFRA (1:600, R&D System, AF-307-NA), mouse anti NR2F2 (COUP-TFII, 1:300, Perseus Proteomics, PP-H7147-00) and guinea pig anti DLX2 (1:1000, from Dr Kazuaki Yoshikawa) [29, 39]. For SOX10 (goat anti-SOX10), HOPX (rabbit anti-HOPX) and PDGFRA (goat anti-PDGFRA) triple immunostaining, we first performed SOX10 immunostaining as it is expressed in the nucleus. After incubation of second antibodies against goat, HOPX and PDGFRA double immunostaining was then performed using the standard protocol as described above.

### scRNA-Seq Datasets Used in This Study

1. Published scRNA-Seq data from 33,976 cells from the mid-gestation human neocortex (3 female donors at GW17, GW17 and GW18, and 1 male donor at GW18) [36] (the accession number is dbGaP: phs001836.) were downloaded from the website https://www.ncbi.nlm.nih.gov/projects/gap/cgi-bin/study.cgi?study_id=phs001836.v1.p1
  Fig. 2 in this study was generated using an online interface at http://geschwindlab.dgsom.ucla.edu/pages/codexviewer
2. Published scRNA-Seq data from 3,355 EGFR-immunopanned cells from the frontal lobe of the human cerebral cortex at GW21-GW26 [35] were downloaded from https://bigd.big.ac.cn/gsa-human/ The accession number for the raw sequencing data is GSA: HRA000348. The accession number for the processed data is GEO: GSE144462.

### scRNA-Seq Datasets Analysis

Strict quality control of cells is required for scRNA-Seq analysis, which has been described in our previous study [6]. For analysis of human scRNA-Seq data from different stages, the pre-processing raw sequencing data from 3,355 human frontal cortical EGFR^+^ cells at GW21–26 (GSE144462) [35] were analyzed by Seurat 3.2.2.9007. Preprocessed data was normalized by log-normalization using a scale-factor of 10,000. The top 2,000 variable genes were identified by the variance stabilizing transformation method, and subsequently scaled and centered. We calculated cell cycle phase scores based on canonical markers [40], and regressed these scores during pre-processing. Principal components analysis was performed for dimensional examination using the ‘elbow’ method. The first 20 dimensions showed the majority of data variability. Therefore, UMAP dimensional reduction was performed on the first 20 dimensions. For cluster annotation, the most comprehensive and reliable cell type markers were sought through extensive literature review [6]. Differentially expressed genes (DEGs) of compatible clusters were identified using following parameters: fold-change-threshold of log (0.25) and with min.pct = 0.25.

### Image Acquisition and Cell Counting

Immunohistochemistry staining images were acquired using Olympus VS120 Automated Slide Scanner. Images for quantitative analyses were acquired using Olympus FV1000 confocal microscope system. Cell counting was performed on Z-stack confocal images. Briefly, the confocal images were imported into Photoshop CC (Adobe Systems Incorporated). We divided the human dorsal and dorsal-lateral neocortex into different number of bins with equal size (250 μm length and 125 μm width in 60 μm thick sections) spanning the cortical VZ, ISVZ (inner subventricular zone), IFL (inner fiber layer) [41], OSVZ [42], IZ (intermediate zone), SP (subplate), CP (cortical plate) and MZ (marginal zone). Cortical layers were delineated by DAPI staining. Labeled cells within each bin were manually counted by using a cell-counter and switching between the red, green, and blue channels in Photoshop. Three representative images of the dorsal or dorsal-lateral neocortex from randomly sampled sections were counted at each age (GW18 and GW23).

### Statistics

Numbers of cells counted per bin in the human dorsal or dorsal lateral cortical VZ, ISVZ, IFL, OSVZ, IZ, SP and CP were shown as mean ± SEM. There were no statistical comparisons performed on cell counts in the present study.

## Results

### scRNA-Seq Analysis Reveals Human Cortical tRG Lineage at GW21-GW26

**Fig. 1:**
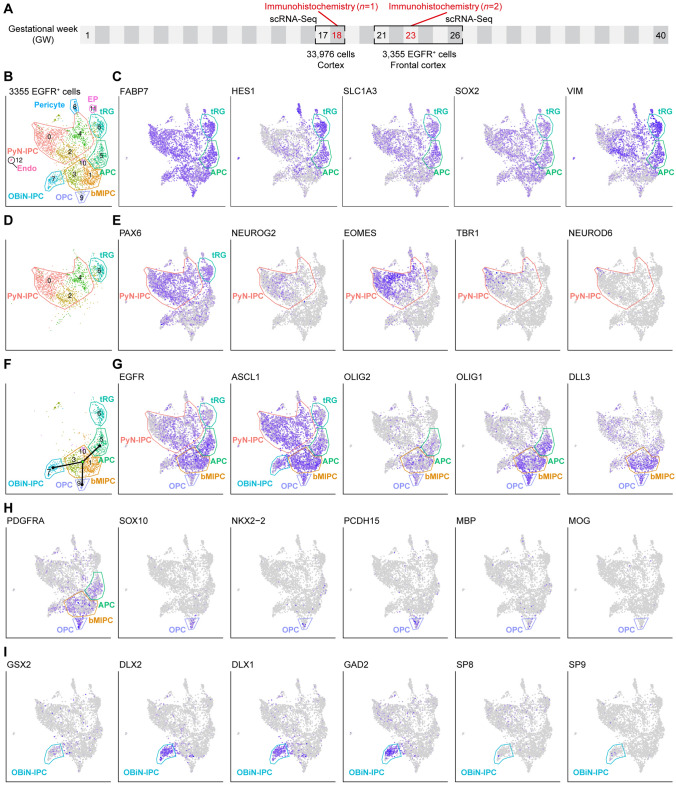
Evidence for cell lineage of the cortical tRG in the human brain. **A** Schematic diagram of the experimental design for this study (also see Materials and Methods). **B** Re-analysis of scRNA-Seq data from 3,355 frontal cortical EGFR^+^ cells at GW21-GW26 [35] revealed the molecular identity of cortical tRG and their progeny (PyN-IPCs, bMIPCs, OPCs, APCs, OBiN-IPCs and EP-ependymal cells). Note that pericytes and endothelial (Endo) cells also expressed EGFR. **C** tRGs and APCs expressed RGC general markers *FABP7, HES1, SLC1A3, SOX2*, and *VIM*. **D, E** *EGFR*^*+*^*ASCL1*^+^*PAX6*^*+*^ cortical tRGs generated *EGFR*^*+*^*ASCL1*^*+*^*PAX6*^*+*^*EOMES*^*+*^ PyN-IPCs. Note that very few *NEUROD6*^+^ immature PyNs expressed *EGFR*. **F–I** Cortical tRGs also generated *EGFR*^*+*^*ASCL1*^*+*^*OLIG2*^*+*^*OLIG1*^*+*^*DLL3*^*+*^ bMIPCs, which in turn gave rise to OPCs (**H**), APCs (**C, G**) and OBiN-IPCs (**I**) (Slingshot unsupervised pseudo-time analysis in **F**). Note that *PDGFRA* was also expressed in a subpopulation of bMIPCs and APCs (**H**). Also note that OBiN-IPCs expressed *GAD2* earlier than *SP8* and *Sp9* (**I**).

Our recent study has demonstrated that the presence of EGFR^+^ progenitors in the E16.5 mouse cortex is a strong signal for the onset of cortical gliogenesis (and OBiN genesis) [6]. To investigate whether a similar process may be occurring in humans, we studied the molecular identity of human cortical EGFR^+^ cells using published scRNA-Seq data. First, we analyzed data from 3,355 EGFR-immunopanned cells obtained from GW21-GW26 human frontal cortex (Fig. 1A) [35]. 13 clusters were recognized (C0-C12) using t-distributed stochastic neighbor embedding (tSNE) and Seurat clustering [43] (Fig. 1B). We identified the identity of the cell types in these clusters based on gene expression. To our surprise, we found that cluster 6 contained cortical tRGs (see below). Furthermore, we obtained evidence that these tRGs (cluster 6) may be lineally related to the PyN-IPC clusters (C4, C2 and C0, 1,531 cells, 46% of EGFR^+^ cells) and bMIPC clusters (C10, C3 and C1) (Fig. 1B), as they all expressed EGFR and ASCL1 (Fig. 1G). Other major cell types were APCs, OPCs and OBiN-IPCs. Consistent with previous analysis, immature ependymal cells (EP), pericytes and endothelial (Endo) cells also expressed EGFR (Fig. 1B), but the authors did not recognize EGFR expression in the cortical tRG [35]. Below, we focus on the molecular features of the tRG, PyN-IPC, bMIPC, APC, OPC and OBiN-IPC cell types.

Cortical RGCs in late embryogenesis express molecules that are typical of the astrocyte lineage cells (including APCs, immature astrocytes and mature astrocytes) [2, 6]. Indeed, our analysis revealed that human cortical tRGs expressed more than 50 genes that are astrocyte-lineage markers (Fig. 1C, Fig. S1), including RGC markers *FABP7, HES1, SLC1A3, SOX2* and *VIM* (Fig. 1C), and astrocyte markers*, ALDOC, AQP4, DBI, GFAP, GLUL, S100B, SLC1A2 and SOX9* etc. (Fig. S1A–G). Notably, *HOPX, FAM107A, LIFR* and *TNC*, markers of vRG and oRG [27, 44, 45], were also expressed by tRGs and APCs (Fig. S1). In addition, FGF and BMP signaling pathways were activated in tRGs and APCs (Fig. S2A, B). tRGs had distinctive properties based on their expression of *ANXA1, ANXA2, APOE, CD38, CRYAB, CXCL12, GLI3, GPX3, MT1E, PDGFD, PDGFRB* and *TMEM47*; these genes were not expressed by APCs at GW21-GW26 (Fig. S2C, D). On the other hand, *CXCL14, DIO2, DUSP6, SDC3, SPARCL1, SPRY1, OLIG2, OLIG1,* and *ID1* were mainly expressed in APCs but not in tRGs (Fig. 1G; Fig. S2B, E). At GW21-GW26, tRGs did not express *ACSBG1, ALDH1L1, FBN2, HS6ST1, HS6ST2* and *NOG* (Fig. S2F); some of these genes are markers of oRG [44].

In general, EGFR was weakly expressed in tRGs and PyN-IPCs (*PAX6*^*+*^*, EOMES*^*+*^ and *NEUROG2*^*+*^), and its expression was downregulated in immature PyNs because very few *NEUROD6*^*+*^*TBR1*^*+*^ PyNs expressed EGFR (Fig. 1D–G). Based on the expression of specific marker genes across major cell types, we deduced likely continuities between specific cell types and probable developmental lineages. For example, while oRGs never expressed *EGFR* and *ASCL1*, tRGs expressed *EGFR, ASCL1* and *PAX6,* and PyN-IPCs continued to express them (Fig. 1D–G), providing evidence that *EGFR*^*+*^ PyN-IPCs are derived from tRGs. Following similar logic, *PAX6*^*+*^*EGFR*^*+*^*ASCL1*^*+*^*BCAN*^*+*^ tRGs appear to give rise to *EGFR*^*+*^*ASCL1*^*+*^*BCAN*^*+*^ bMIPCs (Fig. 1D–G; Fig. S1A). bMIPCs, expressing higher levels of *EGFR* and *ASCL1* than tRGs, also expressed *OLIG2*, *OLIG1*, *DLL3* and *PDGFRA* (Fig. 1F–H); these genes are typical markers for OPCs. This might be the reason why previous studies termed bMIPC as “Pre-OPCs” or “Pri-OPCs” (IPC types preceding committed OPC) [35, 46–49]. Slingshot analysis [50], predicting a developmental trajectory and pseudo-timeline progression of IPCs, suggest that bMIPCs give rise to OPCs (*ASCL1*^*+*^*OLIG2*^*+*^*OLIG1*^*+*^*PDGFRA*^*+*^*SOX10*^*+*^*NKX2-2*^*+*^*PCDH15*^*+*^) (Fig. 1H), APCs (*EGFR*^*+*^* OLIG2*^*+*^*OLIG1*^*+*^*HOPX*^*+*^*ID1*^*+*^*HES1*^*+*^) (Fig. 1G; Fig. S1C) and OBiN-IPCs (*EGFR*^*+*^*ASCL1*^*+*^*GSX2*^*+*^*DLX2*^*+*^*DLX1*^*+*^*GAD2*^*+*^) (Fig. 1I). Early born OPCs and OBiN-IPCs expressed weak *EGFR* and downregulated its expression very soon, whereas APCs expressed higher level of *EGFR* (Fig. 1G).

Our genetic fate mapping study demonstrated that mouse cortical *Egfr*^*+*^*Ascl1*^*+*^*Olig2*^*+*^*Olig1*^*+*^ bMIPC population give rise to most of the OPCs and APCs in the mouse cortex and a subpopulation of OBiN-IPCs [6]. Thus, our re-analysis of scRNA-Seq from human cortical *EGFR*^+^ cells provides evidence for that mouse and human share the same developmental origins of cortical oligodendrocytes and astrocytes, and cortex-derived OBiNs.

### The Coexistence of vRGs, tRGs and oRGs in the Human Cortex at GW17-GW18

**Fig. 2:**
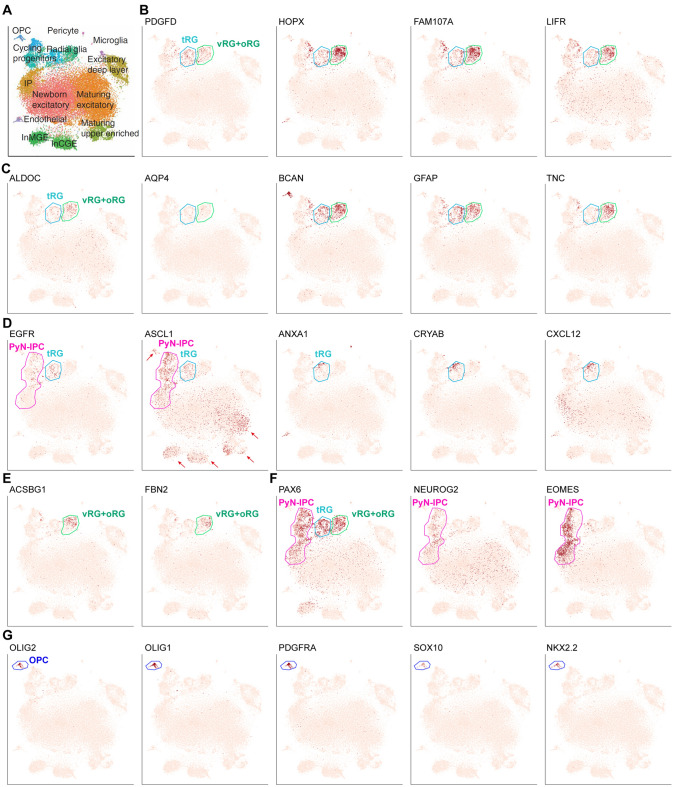
vRGs, tRGs and oRGs coexist in the human cortex at GW17-GW18. **A** scRNA-Seq analysis of 33,976 cortical cells from the mid-gestation human brain (GW17-GW18) [36]. **B, C** Expression patterns of *PDGFD* (vRG and tRG marker), *HOPX*, *FAM107A, LIFR, ALDOC, AQP4, BCAN, GFAP* and *TNC* indicated the coexistence of cortical vRGs, tRGs and oRGs. **D** *EGFR, ASCL1, ANXA1, CRYAB* and *CXCL12* were expressed in tRGs but not in oRGs or vRGs. Note that *EGFR* was weakly expressed in a subpopulation of PyN-IPCs, and *ASCL1* was expressed (indicated by arrows) in a subpopulation of PyN-IPCs, maturing PyNs, and MGE- and CGE-derived cortical interneurons, and MGE-derived OPCs. **E** *ACSBG1* and *FBN2* were expressed in oRGs but not in tRGs. **F** Wide expression of PyN-IPC marker genes (*PAX6*, *NEUROG2* and *EOMES*) indicating activated PyN genesis. **G** Except MGE-derived OPCs, cortex-derived OPC or APC clusters were not identified, suggesting that gliogenesis had not commenced yet in the human cortex at GW17-GW18.

To gain a deeper understanding of the molecular features and the relationships between vRGs, tRGs and oRGs, we next re-analyzed published scRNA-Seq data from the mid-gestation human neocortex (GW17-GW18), which provided a high-resolution transcriptome map of 33,976 cortical cells (Fig. 2A) [36]. vRGs and tRGs expressed *PDGFD* (Fig. S2D) whereas oRGs did not [44, 51]. oRGs expressed higher levels of *HOPX, FAM107A* and *LIFR* than tRGs [27, 44]. Thus, based on expression patterns of *PDGFD, HOPX, FAM107A* and *LIFR*, we show evidence that vRGs, tRGs and oRGs coexist in the human cortex at GW17-GW18 (Fig. 2B).

Here, we propose that vRGs might directly give rise to oRGs and tRGs simultaneously at the end of their final mitosis because vRGs, tRGs and oRGs expressed many shared glial genes, such as *HOPX, FAM107A, LIFR, ALDOC, AQP4*, *BCAN, GFAP* and *TNC* (Fig. 2B, C). Perhaps because tRGs contact the lateral ventricle, they may gradually acquire their unique properties, including expression of *EGFR, ASCL1, ANXA1, CRYAB* and *CXCL12* (Fig. 2D); these genes were not expressed in vRGs or oRGs. On the other hand, oRGs, contacting the pial surface, expressed *ACSBG1* and *FBN2*, which were not expressed in tRGs (Fig. 2E). In the cortex at GW17-GW18, IPCs mainly consisted of PyN-IPCs that expressed *PAX6*, *NEUROG2* and *EOMES* (Fig. 2D, F), suggesting that PyN genesis is the primary cellular product. Indeed, except for the medial ganglionic eminence (MGE)-derived OPCs (Fig. 2G), we did not observe cortex-derived OPC or APC clusters, suggesting that gliogenesis had not yet commenced at GW17-GW18. Notably, while *EGFR* was expressed in tRGs and a subpopulation of PyN-IPCs (progeny of tRG), *EGFR* expression was absent in the OPCs and cortical interneurons that were derived from the MGE and caudal ganglionic eminence (CGE) (Fig. 2D). This demonstrated that *EGFR*^*+*^ cells in the cortex were derived from the cortex itself. *ASCL1*, in contrast, was continuously expressed in a subpopulation of immature PyNs, MGE- and CGE-derived cortical interneurons, and MGE-derived OPCs in the cortex (arrows in Fig. 2D). Consistent with previous report [36], we did not find IPC clusters in the human cortex for generating cortical interneurons, further providing evidence that most if not all human cortical interneurons are derived from the ventral telencephalon [29, 37].

Taken together, by a re-analysis of published scRNA-Seq datasets from human fetal brains at GW17-GW18 and GW21-GW26, we have found that: 1) *EGFR* is expressed in tRGs but not in vRGs or oRGs; 2) *EGFR*^*+*^ tRGs appear to generate *EGFR*^*+*^ PyN-IPCs and *EGFR*^*+*^ bMIPCs; these bMIPCs then give rise to cortical OPCs, APCs, and OBiN-IPCs; 3) *HOPX*, *FAM107A, TNC* and *LIFR* are expressed in tRGs, in addition to vRGs and oRGs; 4) tRGs express numerous hallmarks of cells in the astrocyte lineage.

### Immunohistochemical Identification of tRGs and oRGs in the Human Developing Cerebral Cortex

**Fig. 3:**
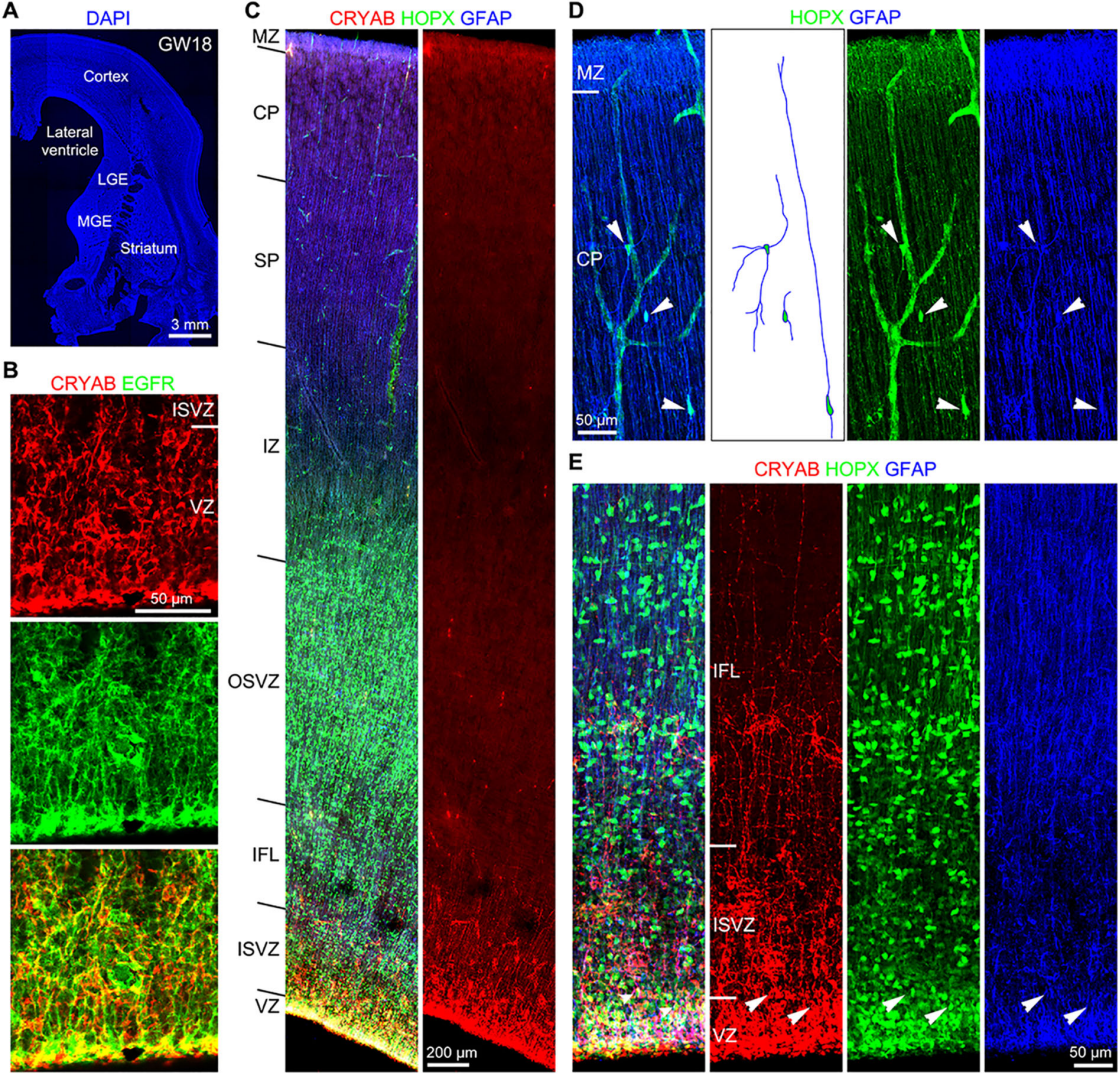
Identification of tRGs and oRGs in the developing human cortex. **A** A coronal section (60 μm thick) of the telencephalon including the cortex, LGE, MGE and striatum (caudate and putamen) of a 18 GW human fetal brain stained for DAPI. **B** CRYAB^+^EGFR^+^ tRGs in the cortical VZ. **C** HOPX, CRYAB and GFAP triple immunostained cortical section at GW18. Note HOPX^+^ cells in the cortical VZ, ISVZ, IFL, OSVZ, IZ, SP and CP, whereas CRYAB^+^ cell somas were mainly in the VZ. **D** HOPX^+^GFAP^+^ astrocyte lineage cells (arrowheads) in the cortical plate. **E** HOPX^+^CRYAB^+^GFAP^+^ tRGs (arrowheads) in the cortical VZ.

To validate the molecular signatures of tRG and oRG identified by the scRNA-Seq analysis, we examined expression of cell type specific marker proteins in fixed cortical sections at GW18 (*n* = 1) and GW23 (*n* = 2) (Fig. 1A). HOPX and GFAP were strongly expressed in somas and/or basal processes of oRGs in the GW18 cortex (Fig. 3A, D, E). At GW23, we observed similar expression patterns of these proteins in the cortex (Fig. S3A–D). On the other hand, double- or triple-immunofluorescence analysis of GW18 cortical sections showed that EGFR, CRYAB, HOPX and GFAP were expressed in tRGs (Fig. 3A–C, E). Consistent with previous observations [27], CRYAB^+^ tRG basal processes terminated in the cortical OSVZ, and thus did not contact the pia (Fig. 3C, Fig. S3A).

CRYAB is a marker for tRGs at GW18 as it is mainly expressed in tRGs. However, at GW23, we observed CRYAB^+^ astrocyte lineage cells that also expressed HOPX and GFAP in the cortical IFL and MZ (Fig. S3B, C) suggesting that CRYAB is more widely expressed in cortical cells at this later fetal stage (Fig. S2C). Taken together, HOPX is expressed in tRGs, in addition to expression in vRGs and oRGs, consistent with our scRNA-Seq analysis (Fig. S1C). This is also consistent with the expression pattern of HOPX in the macaque monkey cortical VZ and OSVZ at E70 and E125 [52] and in the ferret cortical VZ and OSVZ at E36 [53].

### A Proposed Model of the Generation of tRGs and oRGs from vRGs

It has been proposed that human cortical oRGs are generated from vRGs by a process that resembles epithelial-mesenchymal transitions, because oRGs express genes that promote extracellular matrix production, such as *TNC, ITGB5, PTN* and *PTPRZ1* [27, 44]. However, these genes were also expressed in tRGs; furthermore, *TNC, PTN* and *PTPRZ1* were also expressed in vRGs (Fig. 2C, Fig. S1) [36, 44]. Thus, our scRNA-Seq analysis demonstrated that oRGs expressed most of the genes that were also expressed in tRGs (Fig. 1, Figs. S1, S2).

**Fig. 4:**
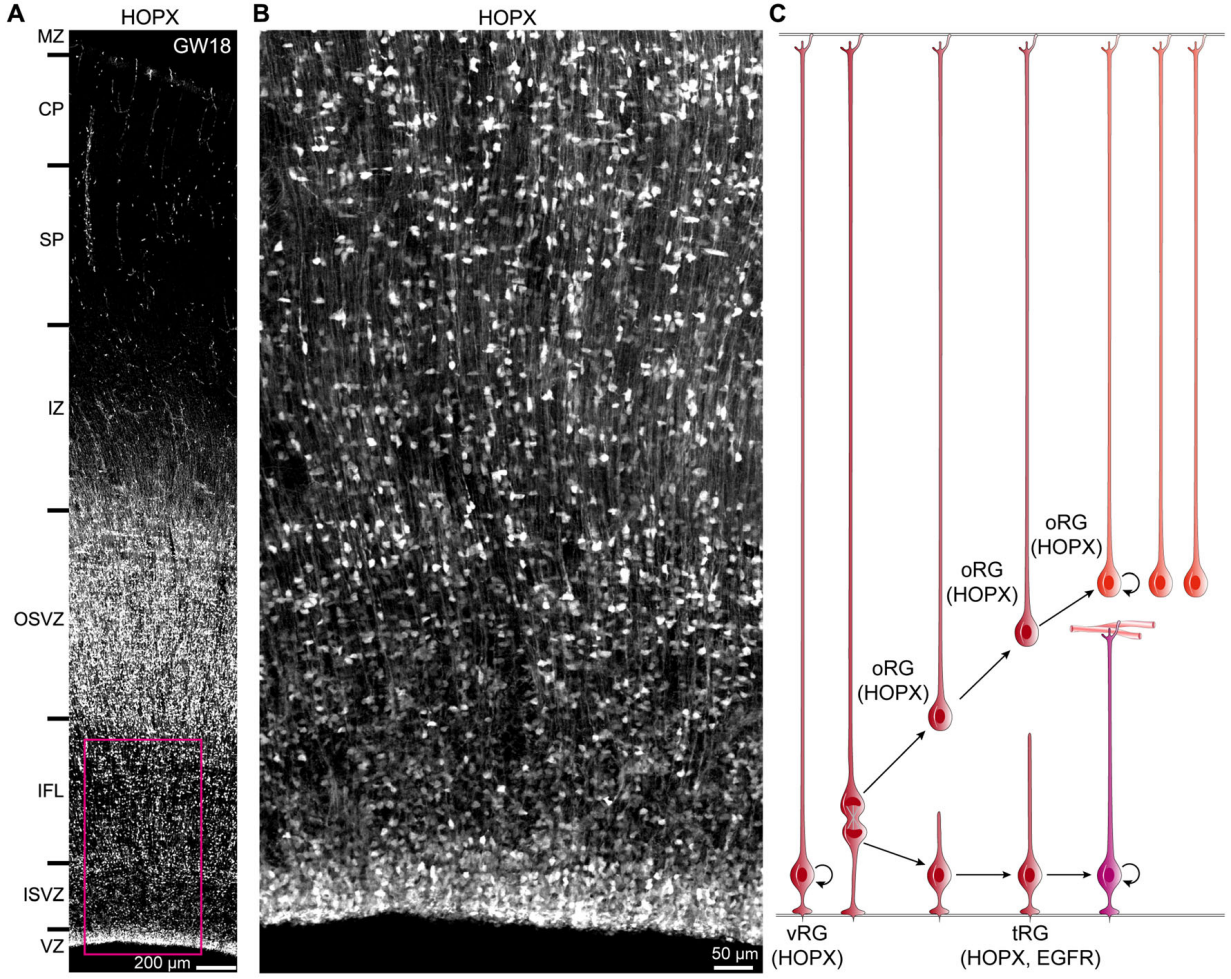
A proposed model for the generation of human cortical tRGs and oRGs. **A** HOPX expression in the human dorsal neocortex at GW18. **B** Higher magnification image of the boxed area in (**A**) showing HOPX^+^ cells in the cortical VZ, ISVZ and IFL. A large number of oRGs with long basal processes are destined to migrate into the OSVZ. **C** Based on HOPX immunostaining results in this study, combined with scRNA-Seq data analysis (Figs. 1 and 2) and time-lapse imaging results [54], we propose that the final mitosis of a HOPX^+^ vRG gives rise to a HOPX^+^ oRG and to a HOPX^+^ tRG. oRGs inherit long basal fibers of vRGs while tRGs inherit apical domains of vRGs. Both oRGs and tRGs can self-renew.

HOPX expression marked multiple progenitor subtypes at GW18. Immunohistochemistry of fixed cortical sections showed HOPX expression in tRGs in the VZ, oRGs in the OSVZ, and in cells in the ISVZ and IFL with long basal processes that appear to be oRGs migrating into the OSVZ (Fig. 4A, B). Previous study using time-lapse imaging have observed that oRGs and tRGs emerge as the daughter cells of horizontally dividing vRGs [54]. Thus, based on HOPX immunostaining results (Fig. 4A, B), combined with scRNA-Seq data (Fig. 1, 2; Figs. S1, S2) and time-lapse imaging analysis [54], we propose that the final mitosis of a HOPX^+^ vRG concomitantly generates a HOPX^+^ oRG and a HOPX^+^ tRG (Fig. 4C). oRGs inherit the long basal fiber of vRGs while tRGs inherit the apical domain of vRGs. Like cortical vRGs, both oRGs and tRGs can self-renew (Fig. 4C).

### HOPX Is also Expressed in Cortical Cells in the Astrocyte Lineage

**Fig. 5:**
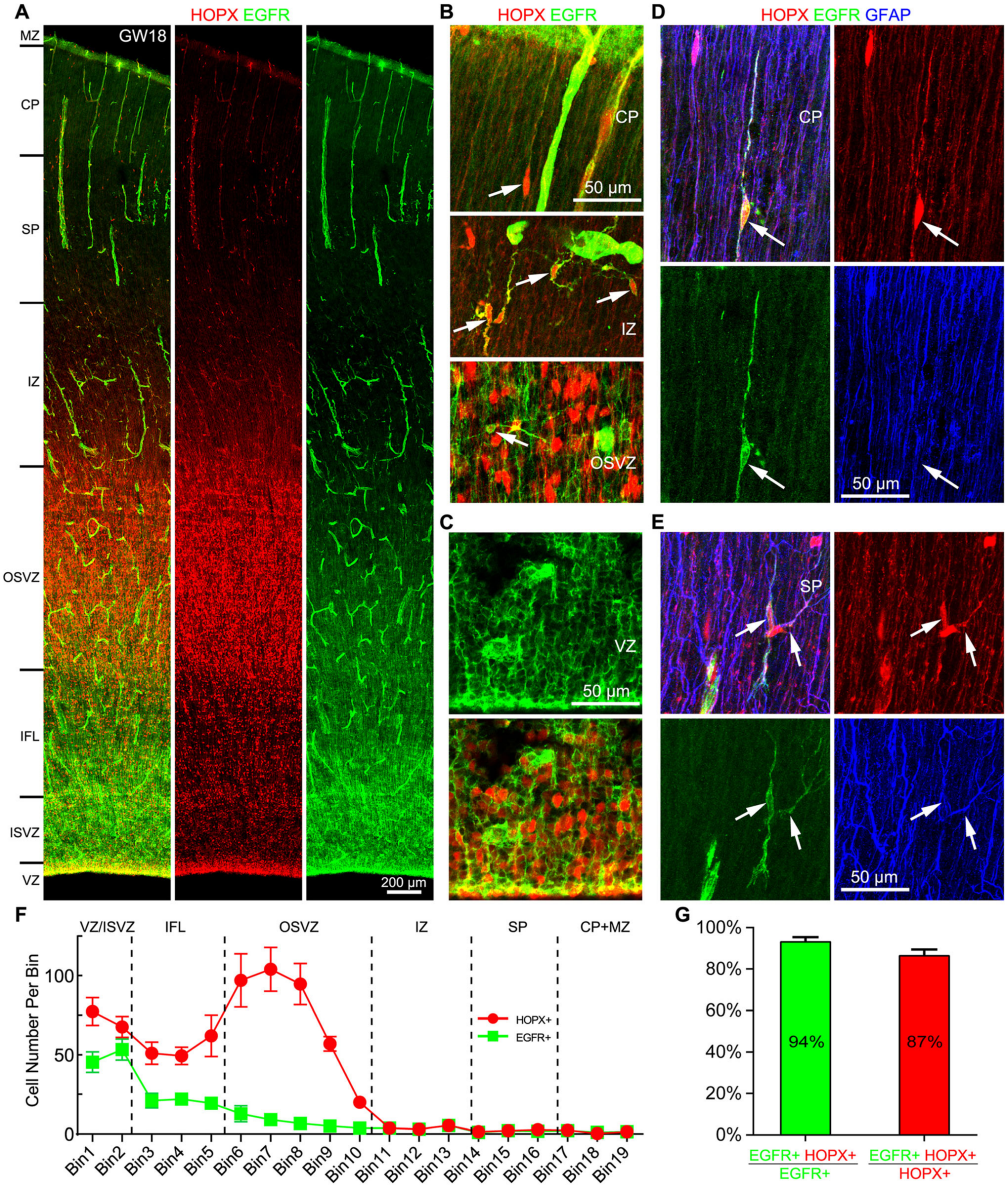
Human cortical tRGs and APCs express HOPX and EGFR. **A** HOPX and EGFR double immunostained GW18 human neocortical section. Note EGFR expression in blood vessels (pericytes and endothelial cells). **B** Higher magnification images showing HOPX^+^EGFR^+^ APCs (arrows) in the cortical CP, IZ and OSVZ. **C** HOPX^+^EGFR^+^ tRGs in the VZ. **D, E** HOPX^+^EGFR^+^GFAP^+^ APCs (arrows) in the cortical CP and SP. **F** Numbers of HOPX^+^ cells and EGFR^+^ cells in the cortex. Note that EGFR^+^ cells were mainly distributed in cortical VZ, ISVZ and IFL. **G** About 94% of EGFR^+^ cells expressed HOPX and 87% of HOPX^+^ cells expressed EGFR in the cortical IZ, SP and CP at GW18.

scRNA-Seq analysis showed that human cortical APCs expressed *EGFR*, *HOPX*, *GFAP*, *OLIG2* and *OLIG1* (Fig. 1G, Fig. S2B, C). We thus examined the expression pattern of EGFR in the GW18 cortex (Fig. 5A). Because endothelial cells and pericytes expressed *EGFR* (scRNA-Seq data, Fig. 1B), cortical blood vessels were EGFR^+^ (Fig. 5A). EGFR was expressed in the cortical VZ and co-labeled with HOPX, further confirming that tRGs expressed EGFR and HOPX (Fig. 5C). However, HOPX^+^ oRGs did not express EGFR (Fig. 5B). From the cortical ISVZ to CP, scattered EGFR^+^HOPX^+^ cells with small somas were observed (Fig. 5B). These cells were APCs, as most of them also expressed GFAP (Fig. 5D, E), consistent with scRNA-Seq analysis (Fig. 1G, Fig. S1B, C). There were ~10-fold more EGFR^+^ cells in the cortical VZ, ISVZ and IFL than that in the OSVZ, IZ, SP and CP (Fig. 5F), suggesting that cortical EGFR^+^ cells are generated in the VZ, and migrate toward the CP. This observation is consistent with previous report [35]. In contrast, there were 4-fold more HOPX^+^ cells in the OSVZ than VZ (Fig. 5F). In the cortical IZ, SP and CP, 94% of EGFR^+^ cells expressed HOPX, and 87% of HOPX+ cells expressed EGFR (Fig. 5G).

**Fig. 6:**
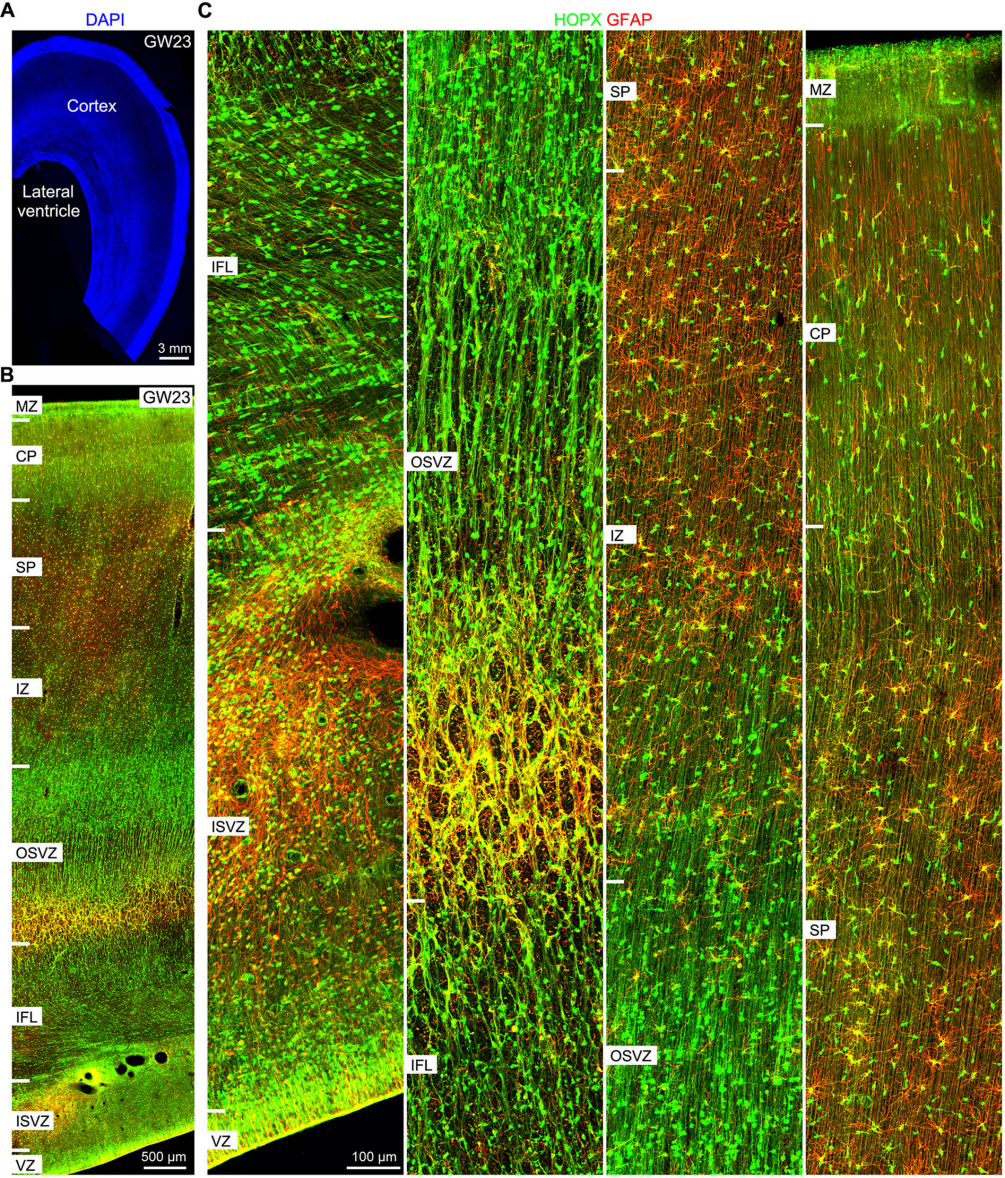
HOPX is expressed in astrocyte lineage cells in the developing human cerebral cortex. **A** A coronal section (60 μm thick) through the human caudal telencephalon at GW23 stained with DAPI. **B** HOPX and EGFAP double immunostained GW23 cortical section. **C** Higher magnification images showed HOPX^+^GFAP^+^ cells; they were tRGs, oRGs and APCs and immature astrocytes.

By GW23, the density of EGFR^+^ cells and HOPX^+^ cells were greatly increased in the cortex compared with GW18 (Figs. S4A and 5A). Again, tRGs expressed EGFR and oRGs did not (Fig. S4B). EGFR^+^HOPX^+^ APCs were widely distributed from the cortical ISVZ to SP, but EGFR was not expressed in most of the HOPX^+^ cells in the cortical CP (Fig. S4B), indicating that EGFR expression is downregulated in HOPX^+^ immature astrocytes. GFAP and HOPX double immunostaining revealed that nearly all HOPX^+^ GW23 cortical cells were GFAP^+^ tRGs, oRGs and astrocyte lineage cells (Fig. 6A–C). The high density of astrocyte lineage cells (HOPX^+^GFAP^+^ cells) in the GW23 cortex suggests a high level of gliogenesis.

**Fig. 7:**
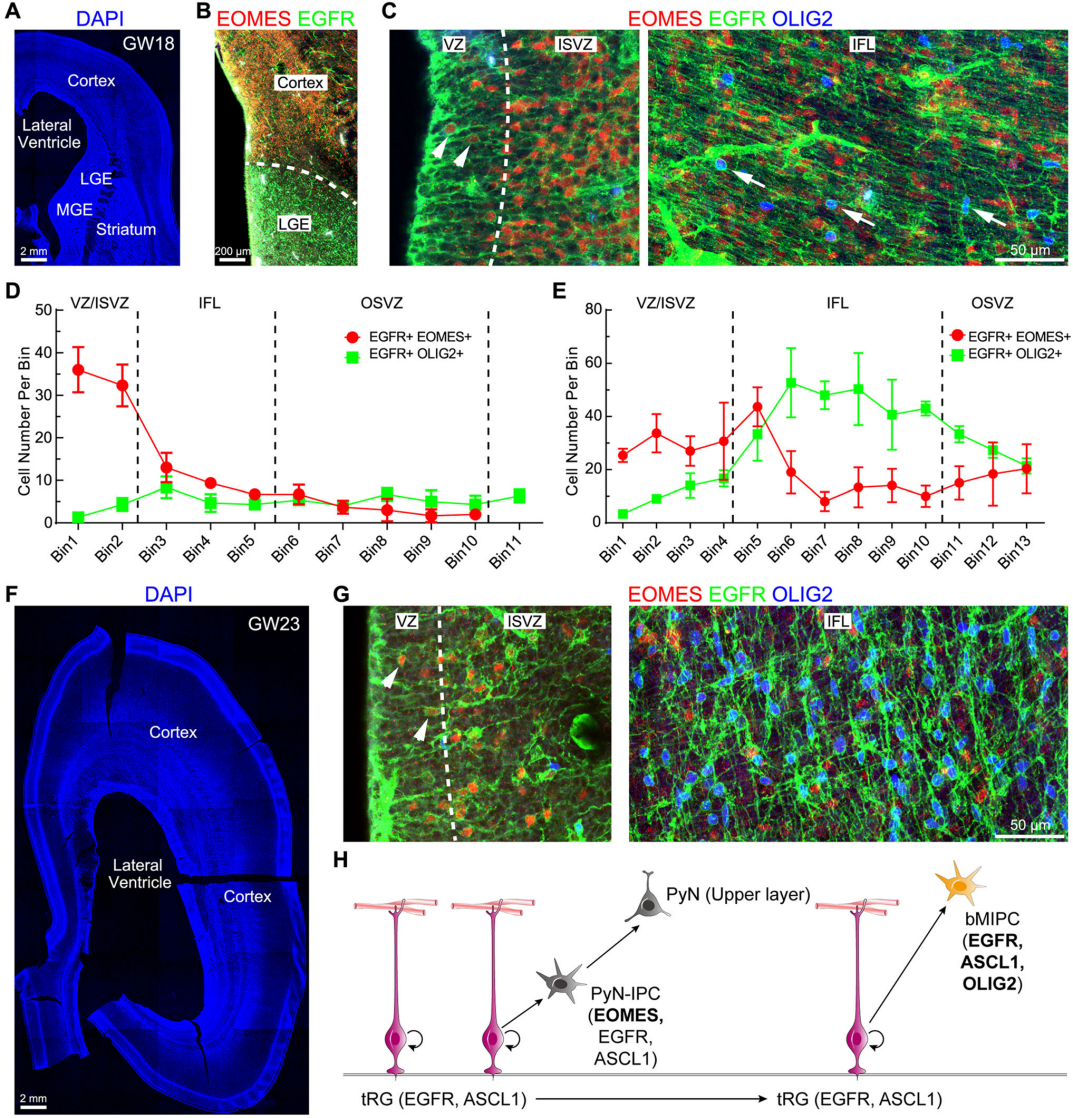
EGFR^+^ tRGs generate EGFR^+^ PyN-IPCs and EGFR^+^ bMIPCs. **A, F** Coronal brain sections (60 μm thick) stained for DAPI. **B** EOMES, EGFR and OLIG2 triple immunostained sections. Note EOMES^+^ cells only in the cortex whereas EGFR^+^ cells in both the cortex and LGE. **C** Higher magnification images showed EGFR^+^EOMES^+^ PyN-IPCs in the cortical ISVZ and EGFR^+^OLIG2^+^ bMIPCs (arrows) in the cortical IFL. **D, E** Numbers of EGFR^+^EOMES^+^ cells and EGFR^+^OLIG2^+^ cells in the VZ, ISVZ, IFL and OSVZ of the human cortex at GW18 (**D**) and GW23 (**E**). **G** Some EGFR^+^EOMES^+^ PyN-IPCs in the cortical ISVZ and a large number of EGFR^+^OLIG2^+^ bMIPCs with a large soma in the cortical IFL were shown. Note that EOMES^+^ cells did not express OLIG2. Also note that EOMES was expressed in some EGFR^+^ SNP like cells (had an apical process extended to the ventricular surface, arrowheads in **C** and **G**). **H** Schematic of human cortical tRGs and their direct progeny. Initially, EGFR^+^ASCL1^+^ tRGs produce EGFR^+^ASCL1^+^EOMES^+^ PyN-IPCs, but later they produce bMIPCs that express higher levels of EGFR, ASCL1 and OLIG2. We propose that EGFR^+^ PyN-IPCs differentiate into upper layer PyNs.

**Fig. 8:**
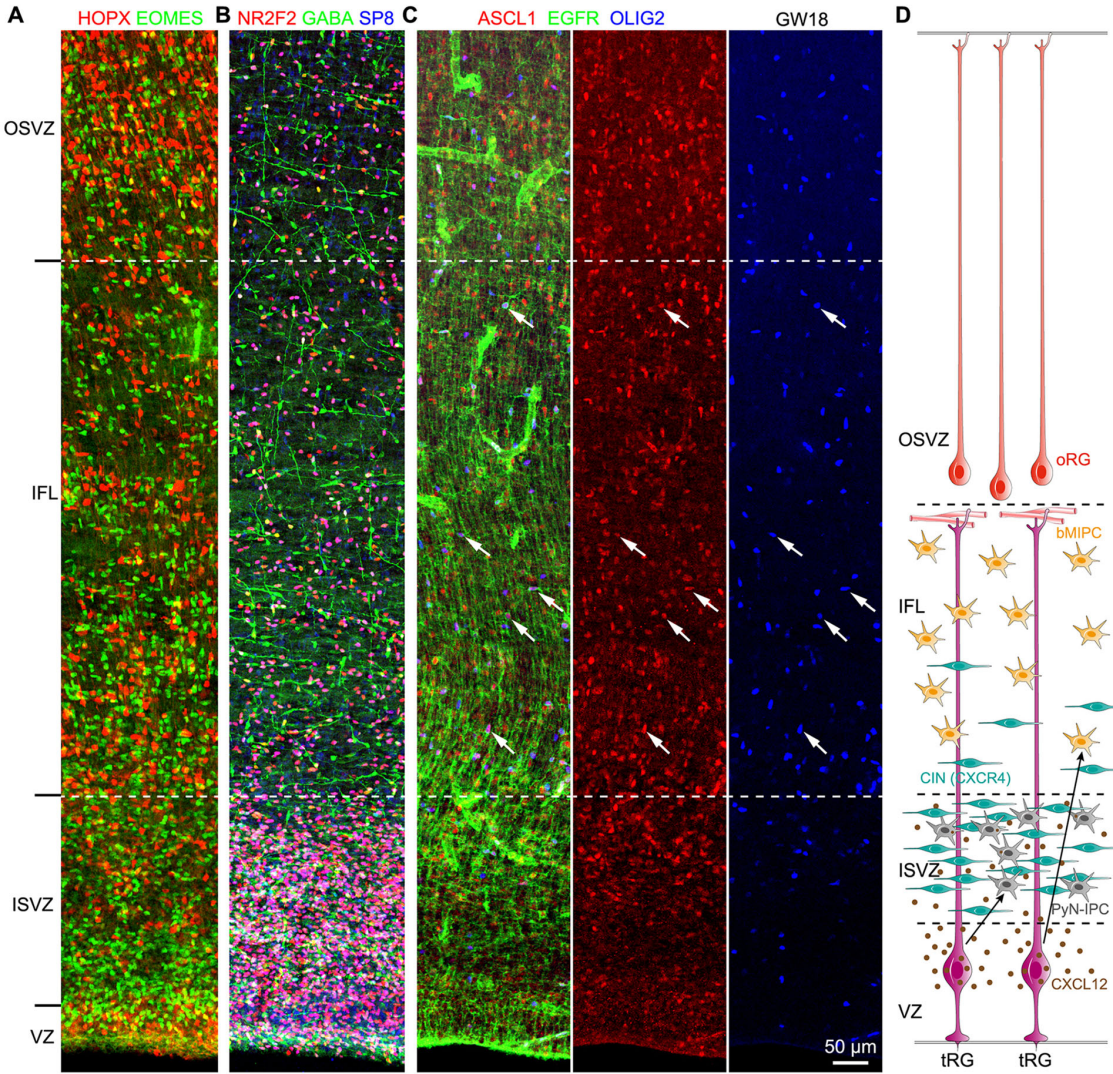
bMIPCs are mainly located in the human cortical IFL. **A, B** The cortical ISVZ contains many EOMES^+^ PYN-IPCs and a large number of migrating CGE-derived cortical interneurons (GABA^+^NR2F2^+^SP8^+^) at GW18. **C** Cortical bMIPCs (EGFR^+^ASCL1^+^OLIG2^+^, arrows) were mainly in the IFL. Note a large number of ASCL1^+^ PyN-IPCs (also EOMES^+^, see Fig. S11) in the cortical ISVZ, IFL and OSVZ. **D** Schema showing that human cortical CXCL12^+^ tRGs are in the VZ, a large number of CXCR4^+^ migrating cortical interneurons (CIN) and tRG-derived PyN-IPCs are in the ISVZ, resulting in the distribution of tRG-derived bMIPCs mainly in the IFL.

**Fig. 9:**
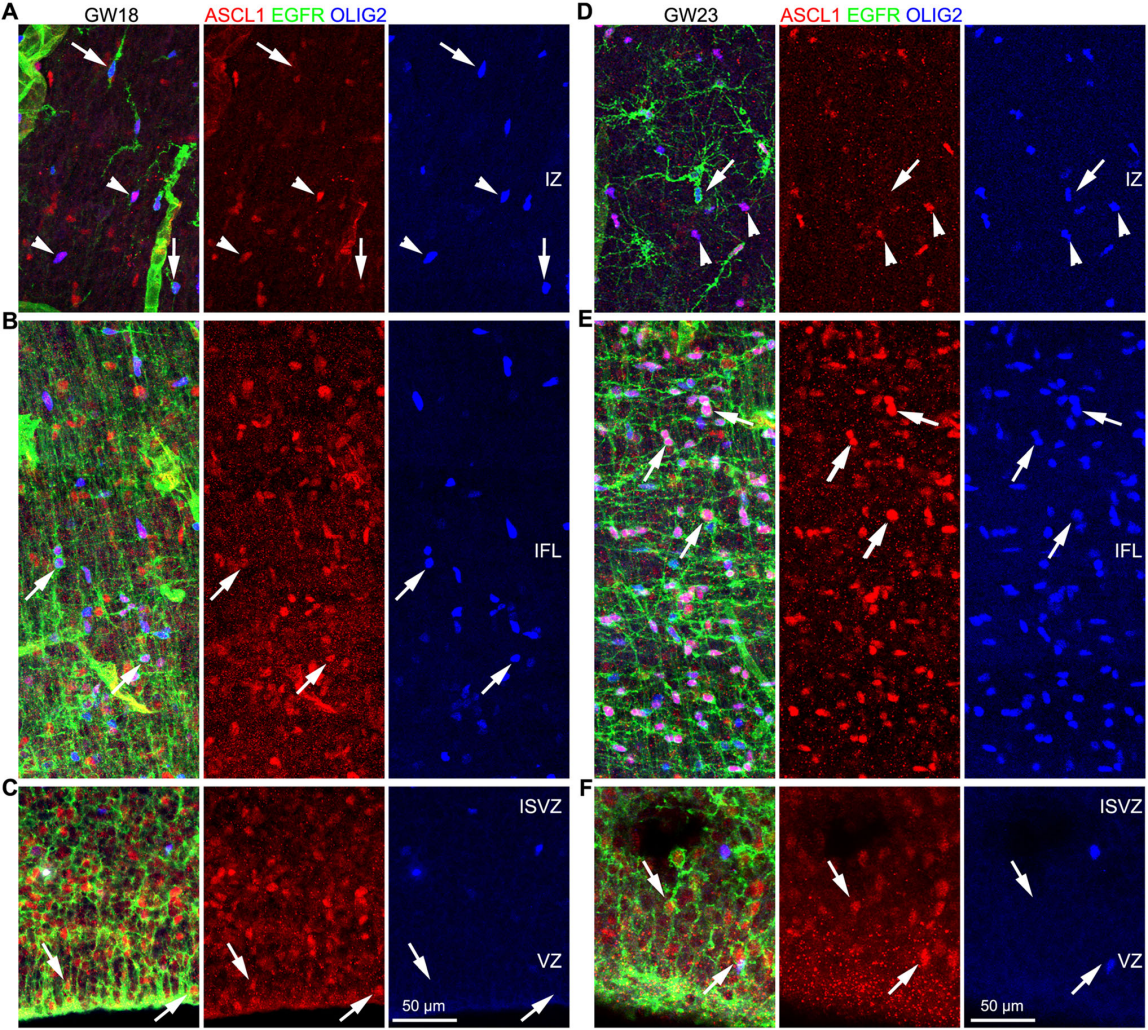
bMIPCs generate cortical OPCs and APCs. **A-F** EGFR, ASCL1 and OLIG2 triple immunostained GW18 and GW23 human cortical sections. tRGs that expressed EGFR and ASCL1 in the cortical VZ were shown (arrows in **C, F**). bMIPCs that expressed EGFR, ASCL1 and OLIG2 in the cortical IFL were shown (arrows in **B, E**). In the cortical IZ, OPCs (arrowheads) expressed ASCL1 and OLIG2, but downregulated EGFR expression, and APCs (arrows) expressed EGFR and OLIG2, but downregulated ASCL1 expression (**A, D**).

To further confirm that HOPX expression marks astrocyte lineage cells, we performed HOPX, SOX10 and OLIG2 triple immunostaining on GW18 cortical sections (Fig. S5A). All SOX10^+^ cells (OPCs) in the cortex expressed OLIG2, but none of them expressed HOPX (Fig. S5B–F), confirming that HOPX is not expressed in OPCs. On the other hand, At GW18, most HOPX^+^ APCs expressed OLIG2 (Fig. S5B–F), consistent with the scRNA-Seq data (Fig. 1G). The ratio of OPCs (SOX10^+^OLIG2^+^ cells) to APCs (HOPX^+^OLIG2^+^ cells) was 2:1 (Fig. S5G), demonstrating that there were more OPCs than APCs in the GW18 cortex. We propose that in the GW18 cortex, most OPCs were derived from the ventral telencephalon [55], whereas most APCs were derived from tRGs, because both APCs and tRGs expressed EGFR and HOPX, and because only a small number of cortical bMIPCs were observed at this stage (Figs. 7, 8, 9). Thus, the earliest macroglial cells in the human cortex are MGE-derived OPCs, followed by cortical tRG-derived EGFR^+^HOPX^+^ APCs.

We also observed that there were more GFAP^+^HOPX^+^ APCs in the ventral cortex than in the dorsal cortex at GW18 (Fig. S6A–C). Thus, in both mouse and human brains, PyNs and glial cells are first generated in the ventral cortex, followed by dorsal and medial cortex [6], suggesting this is a common rule in mammalian brain. Taken together, our immunohistochemistry results demonstrated that in addition to its expression in vRGs, tRGs and oRGs, HOPX marks astrocyte, but not oligodendrocyte, lineage cells in the human cortex.

### Human Cortical tRGs Generate PyN-IPCs and bMIPCs

Our scRNA-Seq analysis provides evidence that human cortical tRGs give rise to PyN-IPCs and bMIPCs (Fig. 1). We thus examined the progeny of tRGs in the GW18 and GW23 human cortex. EOMES and EGFR double immunostaining showed that while EGFR was expressed in both the ganglionic eminences and cortex, EOMES was only expressed in the cortex (Fig. 7A, B). Furthermore, we never observed EOMES^+^ cells (PyN-IPCs) expressing OLIG2 (Fig. 7C, G). Thus, we propose that EGFR^+^ tRGs generate both EGFR^+^EOMES^+^ PyN-IPCs and EGFR^+^OLIG2^+^ bMIPCs (Fig. 7H).

Some EGFR^+^EOMES^+^ cells had an apical process extended to the ventricular surface (Fig. 7C, G), exhibiting morphologies of cortical SNPs. At GW18 and GW23, EGFR^+^EOMES^+^ PyN-IPCs were mainly in the cortical ISVZ, whereas EGFR^+^OLIG2^+^ bMIPCs were mainly in the IFL (Fig. 7D, E). At GW18, only a few EGFR^+^OLIG2^+^ bMIPCs were observed in the cortical IFL (Fig. 7C, D), suggesting that at this stage tRGs mainly produce PyN-IPCs. Thus, there were two germinal zones (niches) for PyN genesis in the GW18 cortex: one in the VZ and the other in the OSVZ. By GW23, there was a high density of EGFR^+^OLIG2^+^ bMIPCs in the IFL (Fig. 7E, G), a high density of astrocyte lineage cells (Fig. 6) and a high density of OPCs (see below, Fig. S10) in the cortex, indicating a high level of gliogenesis. Thus, at GW23 there were two germinal zones: one was the VZ for tRG PyN genesis and gliogenesis, and the other was the OSVZ mainly for PyN genesis from oRGs (see below, Fig. S12, S13). We propose that tRGs and oRGs both generate upper layer PyNs (Fig. 7H, see discussion).

Recently, we have shown that mouse E16.5 cortical RGCs (did not express EGFR) begin to generate EGFR^+^ aMIPCs that differentiate into bMIPCs [6]. To investigate whether mouse EGFR^+^ IPCs also produce PyN-IPCs, like we show in the human cortex, we performed EGFR, EOMES and ASCL1 triple immunostaining on E17.5 and E18.5 mouse cortical sections. We observed EGFR^+^EOMES^+^ PyN-IPCs in the mouse cortical SVZ (Fig. S7A, B), whereas most of the EGFR^+^ASCL1^+^ (also OLIG2^+^) bMIPCs [6] were in the SVZ/IZ border and IZ (Fig. S7A, B). This provides evidence that both human cortical tRGs and mouse cortical RGCs generate EGFR^+^ PyN-IPCs and EGFR^+^ bMIPCs (Fig. 7H and Fig. S7C).

### bMIPCs Are Mainly Distributed in the Human Cortical IFL at GW18-GW23

To further confirm bMIPCs were mainly distributed in the human cortical IFL, we examined cell types in the cortical ISVZ. Immunostaining showed that there were many EOMES^+^ IPCs in the cortical ISVZ (Fig. 8A), but there were many more cortical interneurons, forming a migratory stream, in the cortical ISVZ at GW18 (Fig. 8B). These cortical interneurons expressed GABA, NR2F2 (COUP-TFII) and SP8 (Fig. 8B) suggesting that they are mainly CGE-derived cortical interneurons [29, 37]. Again, very few EGFR^+^ASCL1^+^OLIG2^+^ bMIPCs were in the ISVZ; they were mainly in the IFL (Fig. 8C, D). In the mouse cortex, chemokine CXCL12 is mainly expressed in PyN-IPCs [56, 57], whereas in the human cortex, our scRNA-Seq analysis showed that CXCL12 is mainly expressed in tRGs (Fig. S2C). CXCR4, a CXCL12 receptor, is expressed in nearly all human cortical interneurons [36]. Because CXCL12 is mainly expressed by tRGs in the human cortical VZ, but not by oRGs and their progeny in the OSVZ, cortical ISVZ becomes the main corridor for tangentially migrating cortical interneurons. This may be a key reason for the distribution of bMIPCs mainly in the cortical IFL at GW18-GW23 (Fig. 8D).

### bMIPCs Give Rise to OPCs, APCs and OBiN-IPCs in the Human Cortex

We next examined the lineage of bMIPCs in the human cortex at GW18 and GW23 *in vivo*. First, EGFR^+^ASCL1^+^ tRGs in the cortical VZ were identified (Fig. 9C, F), consistent with scRNA-Seq analysis. Based on proximity, tRGs then generated EGFR^+^ASCL1^+^OLIG2^+^ bMIPCs (Fig. 9B, E). There were only a few bMIPCs in the GW18 cortical IFL, whereas a 10-fold higher density of bMIPCs were observed in the GW23 cortical IFL (Fig. 7C–G, Fig. 9B, E), consistent with the evidence for increased gliogenesis in the GW23 cortex. We suggest that EGFR^+^ASCL1^+^OLIG2^+^ bMIPCs in the cortical IFL give rise to both ASCL1^+^OLIG2^+^ OPCs (Fig. 9A, D), and to EGFR^+^OLIG2^+^ APCs (Fig. 9A, D).

**Fig. 10:**
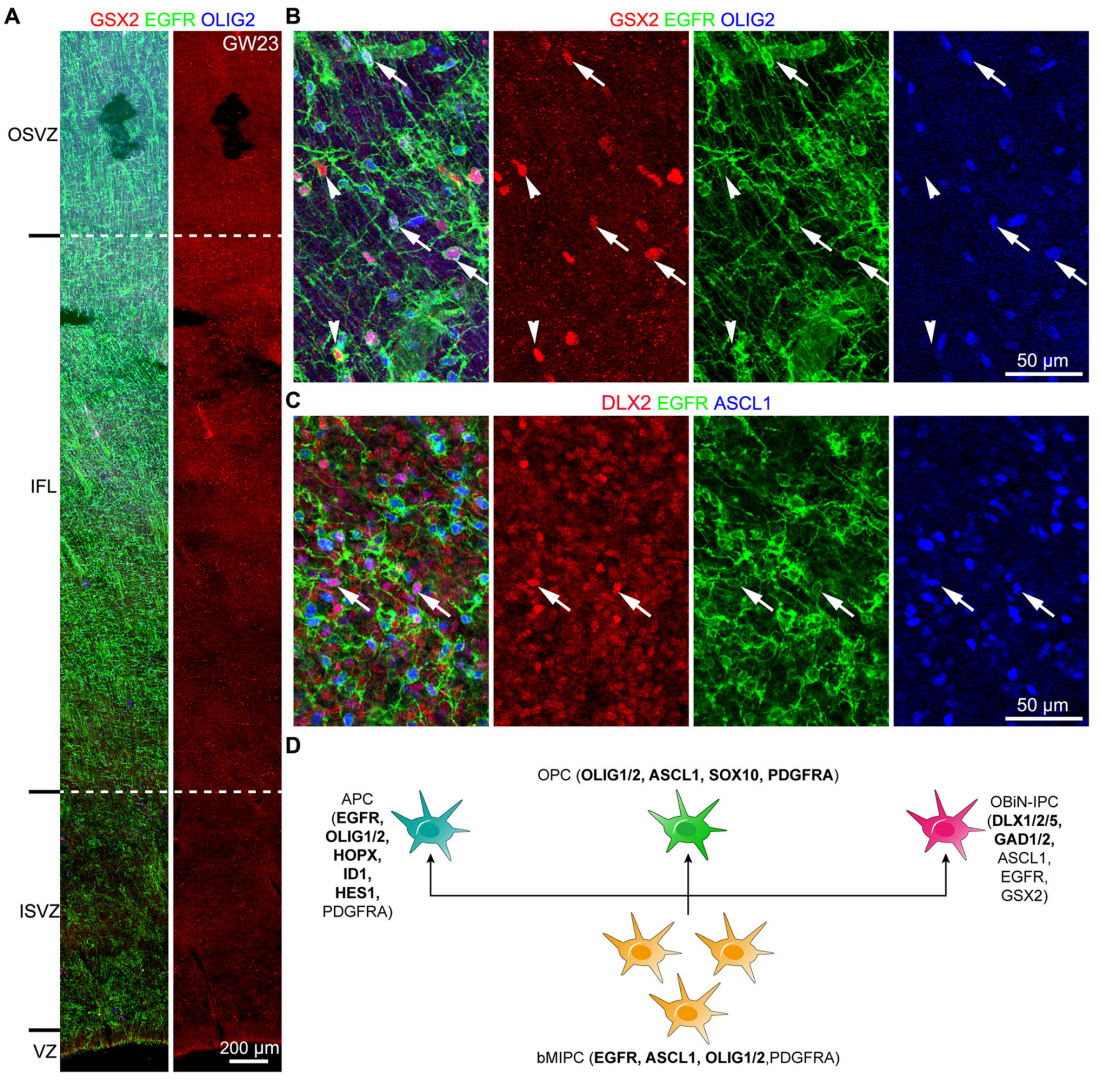
bMIPCs generate OBiN-IPCs in the human cortex. **A** EGFR, GSX2 and OLIG2 triple immunostained GW23 cortical section. Note that GSX2^+^ cells were mainly in the cortical IFL. **B** Higher magnification images showing that a subset of EGFR^+^OLIG2^+^ bMIPCs expressed GSX2 (arrows). We noted that a few EGFR^+^GSX2^+^ cells already downregulated OLIG2 expression (arrowheads). **C** EGFR^+^ASCL1^+^DLX2^+^ cells (OBiN-IPCs) were shown (arrows). **D** Schema summarizing scRNA-Seq analysis and immunohistochemistry results described in the paper. Human cortical bMIPC population give rise to cortical OPCs, APCs and OBiN-IPCs.

We next investigated whether cortical bMIPCs also give rise to OBiN-IPCs as suggested by the scRNA-Seq analysis (Fig. 1F, I). We first examined GSX2 expression in the GW23 cortex; the *GSX2* homeodomain is at the top of the hierarchical gene regulatory network that governs OBiN development in the mouse cortex and dorsal lateral ganglionic eminence (LGE) [39, 58–61]. As expected, GSX2^+^ cells were observed in the GW23 cortex; these cells were also mainly located in the cortical IFL (Fig. S8A-D). We next performed GSX2, EGFR and OLIG2 triple immunostaining, and found some GSX2^+^ cells that expressed EGFR and OLIG2 (Fig. 10A, B). This provided strong evidence that bMIPCs also produced cortical GSX2^+^ cells. We also observed a subset of EGFR^+^GSX2^+^ cells that already downregulated OLIG2 expression (Fig. 10B). GSX2 promotes *DLX* gene expression in the cortex [58–60, 62]. Indeed, some EGFR^+^ASCL1^+^DLX2^+^ cells were identified in the cortical IFL (Fig. 10C), further suggesting that EGFR^+^ASCL1^+^OLIG2^+^ bMIPCs give rise to EGFR^+^ASCL1^+^GSX2^+^DLX2^+^ OBiN-IPCs.

### Human Cortical oRGs Do not Generate OPCs

Two recent studies suggested that cortical oRGs were an additional source of OPCs in the developing human [49] and macaque monkey [52] cortex. The authors found some HOPX^+^ cells expressing EGFR in the cortical OSVZ, therefore suggesting that oRGs generated cortical Pre-OPCs and OPCs [49, 52]. In the human cerebral cortex, Huang *et al*., also found a small number of HOPX^+^ cells expressing PDGFRA, further proposed that oRGs generated Pre-OPCs [49]. However, our results do not support these conclusions. We have identified that HOPX^+^EGFR^+^ cells were cortical tRGs and APCs, but not oRGs (Fig. 5, 6 and Fig. S4).

Next, we examined the expression of PDGFRA in the human cortex at GW18 and GW23. The vast majority of PDGFRA^+^ cells were OPCs in the cortex based on their co-expression with OLIG2 and SOX10 (Fig. S9A–F, Fig. S10). In addition, we found that about 16% of PDGFRA^+^ cells between the GW18 cortical ISVZ and SP expressed HOPX (Fig. S9E, G); they were cortical APCs. We also found that about 16% of PDGFRA^+^ cells in the GW18 cortical IFL and OSVZ expressed neither HOPX nor SOX10 (Fig. S9E, G); they were bMIPCs. These observations were consistent with the scRNA-Seq analysis results (Fig. 1H). Notably, in the GW23 human fetal brain, a large number of OPCs (Fig. S10) and APCs (Fig. 6) were already generated in the cortex, but oRGs were still making PyN-IPCs (see below, Fig. S12, S13), further suggesting that oRGs do not produce OPCs.

Thus, based on the expression patterns of EGFR, ASCL1, PDGFRA, OLIG2, HOPX and EOMES in the cortex, as well as the scRNA-Seq analysis results, we concluded that EGFR^+^ tRG-derived bMIPCs are the major source of cortical OPCs (and APCs) in the human brain. There are a small number of OPCs in the human cortex that are derived from the MGE, but we did not find any evidences supporting the claim that oRGs are an additional source of cortical OPCs.

### PyN Genesis Continues at GW23

There were two germinal zones, VZ and OSVZ, in the GW18 and GW23 cortex. HOPX and EOMES double immunostaining revealed a large number of EOMES^+^ cells in both the cortical ISVZ and OSVZ at GW18; some of them expressed ASCL1 (Fig. S11A–D) [28, 37]. In the GW23 cortex, a large number of EOMES^+^ cells in the OSVZ and ISVZ were observed (Fig. S12A–C), further confirming that the GW23 cortical OSVZ is still in the peak of PyN genesis, whereas cortical VZ is making both PyNs and glia. A recent study reported that the number of EOMES^+^ cells was reduced in the GW19-GW20 cortex and proposed that the production of PyNs ceases in the human fetal cortex after GW20 [63]. However, as indicated above, we observed a large number of EOMES^+^ cells in the GW23 cortex (Fig. S12); all of these EOMES^+^ cells expressed PAX6 (Fig. S13) and none of them expressed OLIG2 (Fig. 7G), suggesting that human cortical PyN genesis indeed occurs at GW23. Our observation was again supported by the scRNA-Seq data: among 3,355 tRG-derived EGFR-immunopanned cells obtained from GW21-GW26 human frontal cortex, there were 1,531 cells belonged to PyN-IPCs (46% of EGFR^+^ cells) (Fig. 1A) [35]. Thus, during GW21-GW26, human cortical tRGs and oRGs are still making PyN-IPCs.

## Discussion

**Fig. 11:**
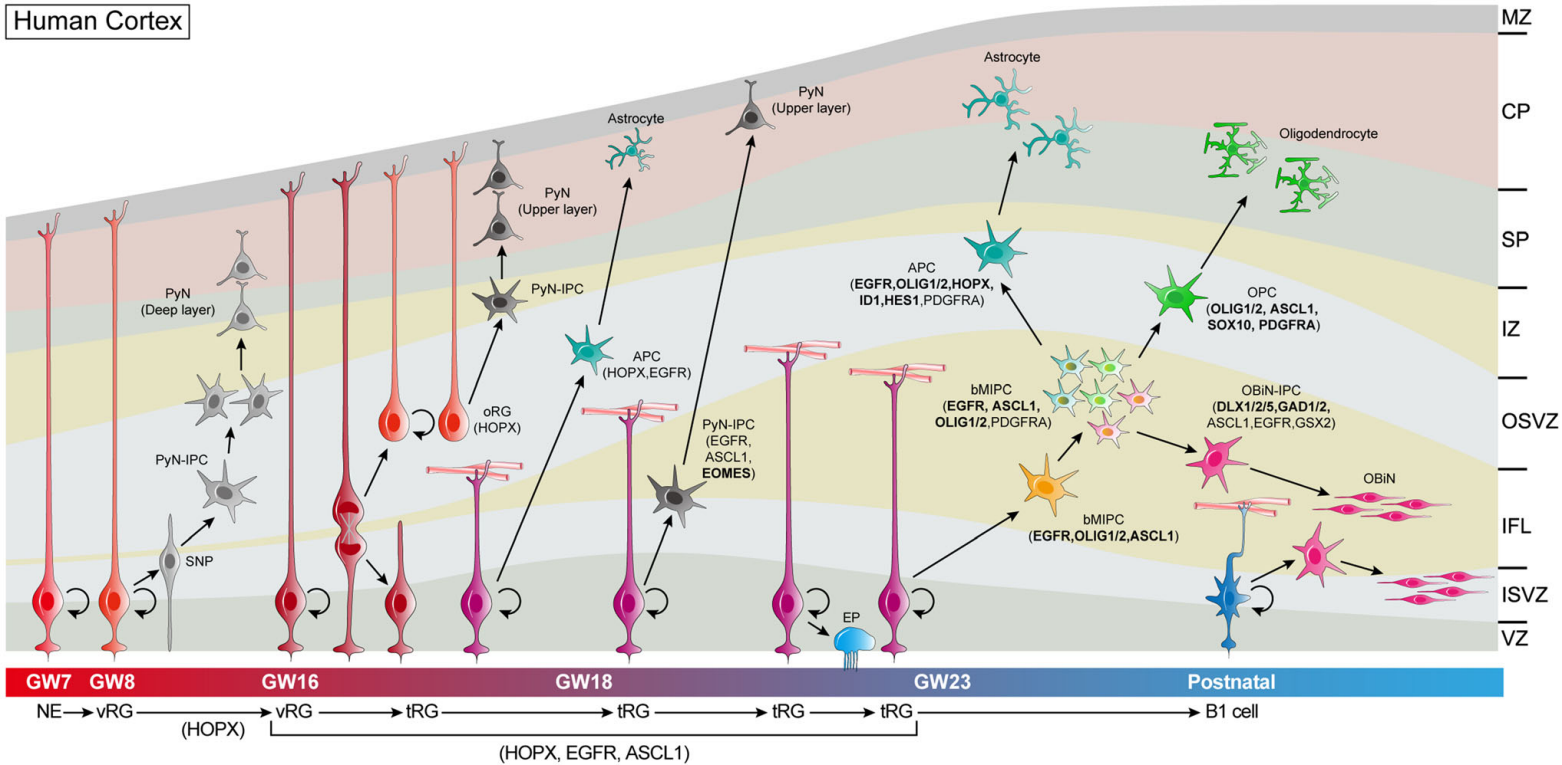
Summary of cortical RGC lineage progression and origins of cortical oligodendrocytes, astrocytes and cortex-derived OBiNs in the human brain. Neuroepithelial cells (NEs) convert into vRGs as the developing human cortex thickens around GW7. During GW8-GW15, vRGs gradually begin to express HOPX, undergo asymmetric cell division to self-renew, and to produce short neural precursors (SNPs). Cortical SNPs, represent transitional cell form between vRGs and PyN-IPCs, differentiate into PyN-IPCs, which exclusively generate deep layer PyNs. Around GW15-GW16, human cortical HOPX^+^ vRGs horizontally divide into a HOPX^+^ oRG and a HOPX^+^ tRG. oRGs inherit long basal fibers of vRGs while tRGs inherit apical domains of vRGs. Both oRGs and tRGs can self-renew. HOPX^+^ oRGs generate upper layer PyNs. tRGs gradually express EGFR and ASCL1 and initially produce EGFR^+^EOMES^+^ASCL1^+^ PyN-IPCs, which also differentiate into upper layer PyNs. Some tRGs may directly transform into HOPX^+^EGFR^+^ APCs; some convert into ependymal cells (EPs). Around GW23, a large number of tRGs generate bMIPCs that express EGFR, ASCL1 and OLIG1/2. bMIPCs undergo several rounds of mitosis to generate OPCs, APCs and OBiN-IPCs in the human cortex. These lineage-restricted IPCs then divide symmetrically to generate cortical oligodendrocytes, astrocytes and OBiNs. In the neonatal human brain, cortical tRGs (B1 neural stem cells) may predominantly generate OBiN-IPCs, but this process only occurs during the first year of life. In the adult human SVZ, very few newly born OBiNs are detected.

Our study has 4 main findings: (1) The molecular identity of the human cortical tRG based on re-analysis of scRNA-Seq datasets; (2) A model of human cortical RGC lineage progression (Fig. 11); (3) Cortical EGFR^+^ tRGs first generate EGFR^+^EOMES^+^ upper layer PyN-IPCs, and then generate EGFR^+^ASCL1^+^OLIG1/2^+^ bMIPCs which in turn give rise to most of the cortical OPCs and APCs, and cortex-derived OBiN-IPCs (Fig. 11). (4) The developmental origins of cortical glial cells (oligodendrocytes and astrocytes) and cortex-derived OBiNs in the human brain is evolutionary conserved based on this study and our previous one in mouse cortex using genetic lineage tracing (Fig. 11) [6].

### RGC Lineage Progression and Gliogenesis in the Human and Mouse Cortex

Cortical RGCs generate different subtypes of cells at different developmental time points [2, 64–66]. Cortical RGCs gradually express more and more hallmarks of astrocytes (Fig. S1, S2), and begin to make bMIPCs, which then generate OPCs, APCs and OBiN-IPCs in the human midfetal cortex. In the mouse, at the end of cortical PyN genesis around E16.5, in the lateral cortex, RGCs begin to produce EGFR^+^ IPCs, which is indicative of the onset of cortical gliogenesis (and OBiN genesis) [6]. However, EGFR^+^ IPCs do not immediately produce bMIPCs, instead, they continue making EGFR^+^EOMES^+^ IPCs. Around E17.5, many mouse cortical RGCs produce EGFR^+^ aMIPCs that differentiate into bMIPCs, which in turn give rise to cortical OPCs, APCs and OBiN-IPCs [6]. Thus, the onset of cortical gliogenesis in the mouse brain involves a sequence of steps. This process is mediated by many molecules, including the secreted signaling proteins BMP, SHH and FGF [6, 62, 67, 68]. Most importantly, we proposed that with EGFR expression and sustained activation of the MEK/MAPK pathway in the mouse cortical VZ/SVZ, cortical IPCs upregulate the expression of identity genes of bMIPCs (*Egfr*, *Olig1/2* and *Ascl1*), OPCs (*Pdgfra* and *Sox10*), APCs (*Id3* and *Aldh1l1*) and OBiN-IPCs (*Gsx2, Dlx1/2/5, Gad2/1* and *Sp8/9*) [69–71].

In the human developing cortex, three types of cortical RGCs have been identified: vRGs, tRGs and oRGs [27, 28, 44]. At GW8-GW15, vRGs mainly generate deep layer PyNs. Around GW15-GW16, human cortical vRGs start to differentiate into oRGs and tRGs. We propose that the commencement of EGFR^+^ tRGs is a strong signal indicating onset of human cortical gliogenesis. Initially, these EGFR^+^ tRGs produce EGFR^+^EOMES^+^ PyN-IPCs, and then they produce bMIPCs. Thus, onset of gliogenesis in the human cortex is also a sequence of steps, but one that takes place over a much longer period than in the mouse cortex. This one reason that we interpret that 46% of EGFR^+^ cells in the GW21-GW26 cortex (scRNA-Seq data) correspond to PyN-IPCs (Fig. 1B). EGFR and EOMES double immunostaining also revealed that most EOMES^+^ PyN-IPCs in the GW18 and GW23 cortical ISVZ expressed EGFR (Fig. 7A–D). It is well established that deep layer PyNs are the first PyNs to be generated [2, 72–75]. We thus propose that tRG-derived EGFR^+^EOMES^+^ PyN-IPCs become upper layer PyNs. Thus, a series of steps are required to generate tRG-derived PyN-IPCs, bMIPCs, OPCs, APCs and OBiN-IPCs.

### Fate Specification of Cortical OPCs, APCs and OBiN-IPCs in the Human Cortex

Human cortical bMIPCs expressed higher levels of *EGFR, ASCL1, OLIG1/2*, and weak *PDGFRA* (Fig. 1F–H). The molecular features of bMIPCs closely resemble OPCs, therefore, many previous studies called bMIPCs as Pre-OPCs or Pri-OPCs [35, 46–49]. These studies, in conjunction with ours, provides strong evidence that as EGFR expression wanes cortical bMIPCs are biased toward OPC fates. Currently, we do not know why EGFR expression must be downregulated in the OPCs, but we hypothesize that it may interfere with PDGFR signaling in promoting OPCs proliferation and survival.

When bMIPCs give rise to APCs in the cortex, APCs maintain *EGFR* and *OLIG1/2* expression, but downregulate *ASCL1* expression (Fig. 1G). This process is prolonged in the human cortex than that in the mouse cortex, partially because the upregulation of *ID3* expression in human astrocyte lineage cells is very late. Indeed, we only observed very few *ID3*^+^ cortical APCs in the human brain at GW21-GW26 (Fig. S2B), but with the upregulation of *ID1, ID2, ID4* and *HES1*, downregulation of *ASCL1* and *OLIG1/2* expression [6, 76, 77], and further downregulation of *EGFR* expression [78], human cortical APCs gradually differentiate into immature and mature astrocytes. It is also worth noting that downregulation of *ASCL1* (protein and mRNA) expression in PyN-IPCs, immature PyNs and cortical interneurons in the human cortex takes a much longer time than that in the mouse cortex (Figs. 1G, 2D). Cortical astrocytes have two origins: bMIPCs are the major source of cortical astrocytes, and translocating cortical RGCs give rise to a smaller population of cortical astrocytes, as observed in the developing macaque monkey and ferret cortex [4, 79, 80]. We proposed that a subset of all three types of human cortical RGCs: vRGs, tRGs and oRGs, may directly transform to APCs in the human cortex. Currently, we do not know many differences between these two groups of astrocytes, but we do know transforming cortical RGC-derived APCs appear earlier in the cortex than bMIPC-derived APCs (Fig. 5A–E).

In the mouse cortex, we have identified that *Egfr*^+^*Id3*^+^ cells are earliest APCs, which then upregulate *Aldh1l1* expression in nearly all astrocytes [6]. *Aldh1l1* is expressed in mouse cortical RGCs in late embryogenesis, but downregulated in bMIPCs and re-upregulated in APCs and strongly expressed in immature and mature astrocytes [6, 81, 82]. In the developing human cortex, however, both *ID3* and *ALDH1L1* expression are delayed in astrocyte lineage cells (Fig. S2B, F). Instead, the expression of HOPX and GFAP in human cortical APCs is much earlier than that in the mouse cortex (Figs. 5, 6). Indeed, mouse GFAP protein is not widely expressed in the mouse cortical RGCs until P0, but 2.2-kb human-GFAP promoter driving expression of EGFP can be detected in the mouse cortical RGCs as early as E13.0 [83]. Thus, HOPX and GFAP are reliable markers to characterize astrocyte lineage cells in the prenatal and postnatal human cortex.

OBiN-IPCs and bMIPCs have many molecular differences, but they are still likely to be lineally related (Fig. 1F). *EGFR*^*+*^*ASCL1*^*+*^*OLIG1/2*^*+*^ bMIPCs give rise to *EGFR*^*+*^*ASCL1*^*+*^*GSX2*^*+*^*DLX1/2*^*+*^ OBiN-IPCs that downregulate *OLIG1/2* expression (Fig. 1I). We have identified a gene regulatory network, *Gsx1/2* – *Dlx1/2/5/6* – *GAD2/1/Sp8/Sp9* – *Tshz1* – *Prokr2*, which governs OBiN development [39, 84–88]; mutations of some of these genes result in human Kallmann syndrome with abnormal olfactory function (anosmia or hyposmia) [89, 90]. Thus, initiation of cortical expression of GSX2 and DLX2 is crucial for the generation of OBiN-IPCs [39, 58, 59]. However, onset of OBiN genesis in the cortex is also a sequence of steps, as *Gsx2* and *Dlx1/2* expression does not represent an irreversible state of OBiN commitment; a small number of *Gsx2*^*+*^ and/or *Dlx2*^*+*^ IPCs also give rise to cortical macroglial cells [62, 91]. With the accumulation of DLX1/2 proteins and the induction of *Dlx5/6, GAD1/2* and *Sp8/9*, bMIPCs differentiate into OBiN-IPCs, and their interneuronal fate is finally determined.

In the postnatal human cortical parenchyma, OPCs and APCs continue to divide [92]. Neural stem cells (type B1 neural stem cells, like tRGs) in the cortical SVZ may predominantly generate OBiN-IPCs [93] (Fig. 11), but this process only occurs during the first year of life [94]. In the adult human SVZ, newly born OBiNs are rare [95, 96], suggesting that neurogenesis is not present in the adult human brain [97].

### Why Does Our Human Brain Need a Cortical OSVZ?

The findings described in this and other studies [24, 30, 98] raise several interesting theoretical issues. For example, why does the human brain need a cortical OSVZ? One likely reason is that with the evolutionary expansion of our neocortex it needs more PyNs. Indeed, it was reported that human cortical neurogenesis extends into the third trimester [99] (also see Fig. S12, S13). Another critical reason may be that the expanded neocortex needs more glial cells, as early as possible, because they have multiple functions in the developing cortex [100, 101].

The human neocortex has two germinal zones. oRGs in the OSVZ mainly produce cortical upper layer PyNs, from GW16 to the third trimester. In contrast, the main function of tRGs in the cortical VZ is to generate upper layer PyNs first, and then generate cortical macroglial cells (and OBiNs). Thus, the human neocortex can simultaneously generate PyN-IPCs in the OSVZ and glial-IPCs (bMIPCs) in the ISVZ, so there is no way one can interfere with the other. According to this model, the specification of cortical RGCs (tRGs) may take place only in proximity to morphogens (e.g., SHH and FGF) in the cortical VZ that are implicated in glial and OBiN cell type specification [62, 67, 68, 102–105].

Taken together, these findings extend our understanding of the origins, lineage relationships, timing of differentiation, and molecular properties of cortical oligodendrocytes, astrocytes, and cortex-derived cortex-derived OBiNs in the human fetal brain. This also provides important insights into the origins of malignant glioma cells.

## Supplementary Information

Below is the link to the electronic supplementary material.

Supplementary file1 (PDF 9507 kb)
