# Supplementary material for: Developmental Origins of Human Cortical Oligodendrocytes and Astrocytes
Source: Neurosci Bull. 2021 Aug 10;38(1):47–68. doi: 10.1007/s12264-021-00759-9 (PMC8783027; doi:10.1007/s12264-021-00759-9)
Supplement: Supplementary file 1 — Supplementary file1 (PDF 9507 kb) [file 12264_2021_759_MOESM1_ESM.pdf]

Supplementary Materials

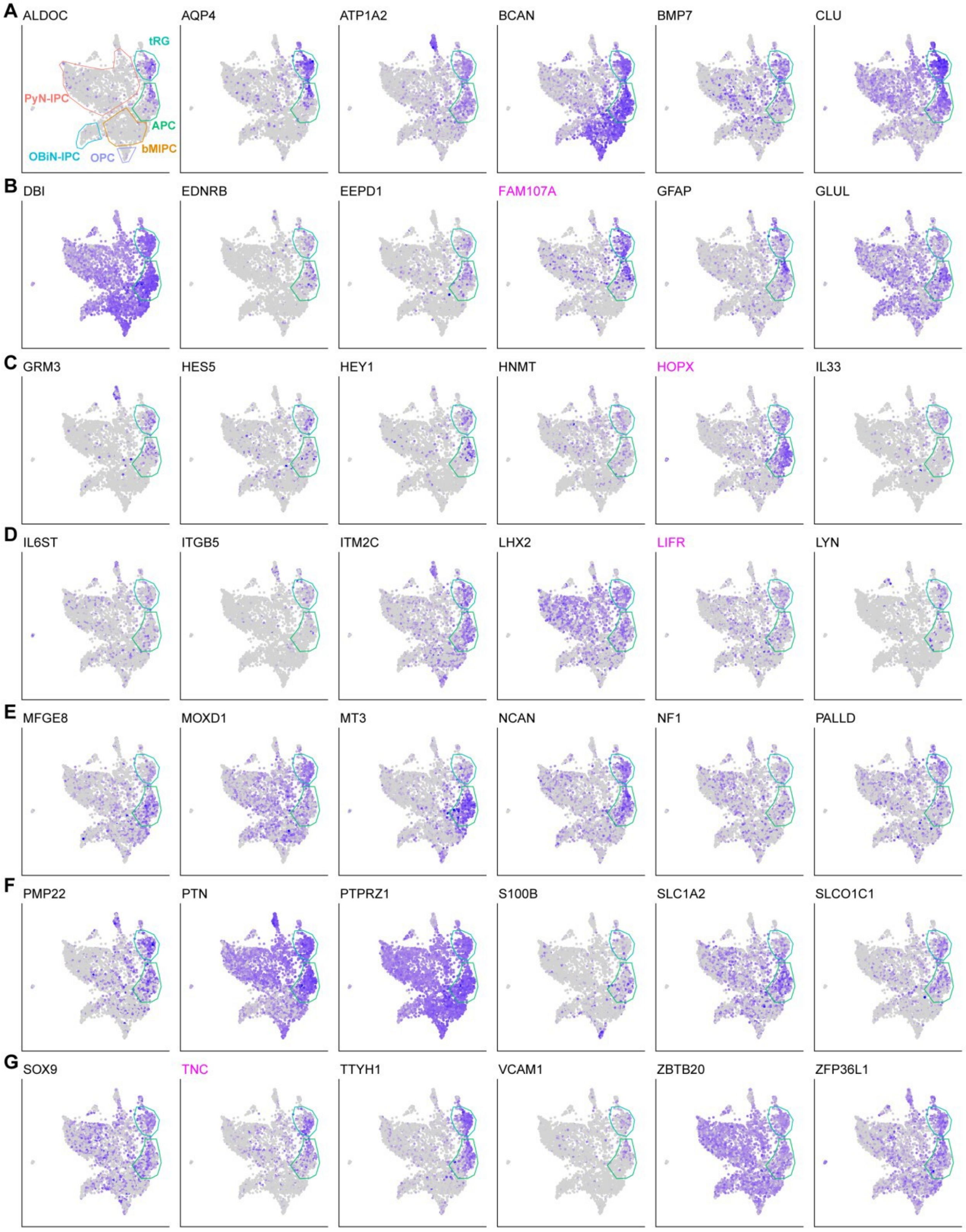

Fig. S1 The molecular identity and glial nature of the human cortical tRG. **A–G** Re-analysis of scRNA- Seq data from 3,355 frontal cortical EGFR$^+$ cells at GW21-GW26. EGFR$^+$ human cortical tRGs at GW21- GW26 expressed astrocyte lineage cell (including APC, immature astrocyte and mature astrocyte) marker genes. This observation is consistent with the notion that cortical RGCs are characterized by the expression of numerous molecules that are typical of astrocytes in late embryogenesis. Note that most of these genes were expressed in tRGs, downregulated in bMIPCs, and then re-upregulated in APCs.
Also note the expression of *HOPX, FAM107A, LIFR* and *TNC* in cortical tRGs.

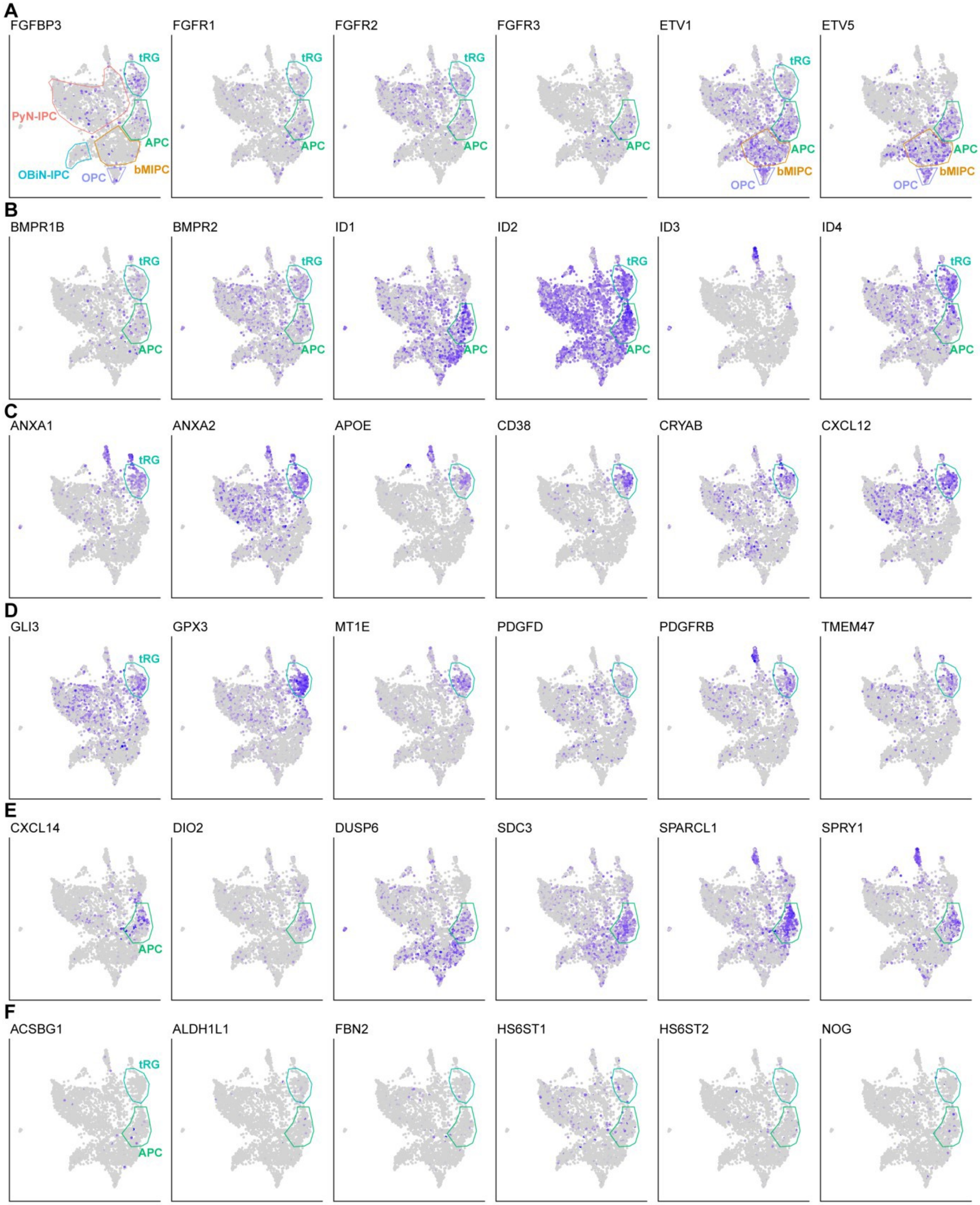

Fig. S2 Re-analysis of scRNA-Seq data from 3,355 frontal cortical EGFR$^+$ cells at GW21-GW26 reveals gliogenic potential of cortical tRGs. **A, B** FGF and BMP signaling pathways were activated in human cortical tRGs and APCs. **C, D** Genes that were mainly expressed in cortical tRGs but not in APCs. **E** Genes that were expressed in APCs but not in cortical tRGs. **F** Human cortical tRGs did not express *ACSBG1, ALDH1L1, FBN2, HS6ST1, HS6ST2* and *NOG* at GW21-GW26.

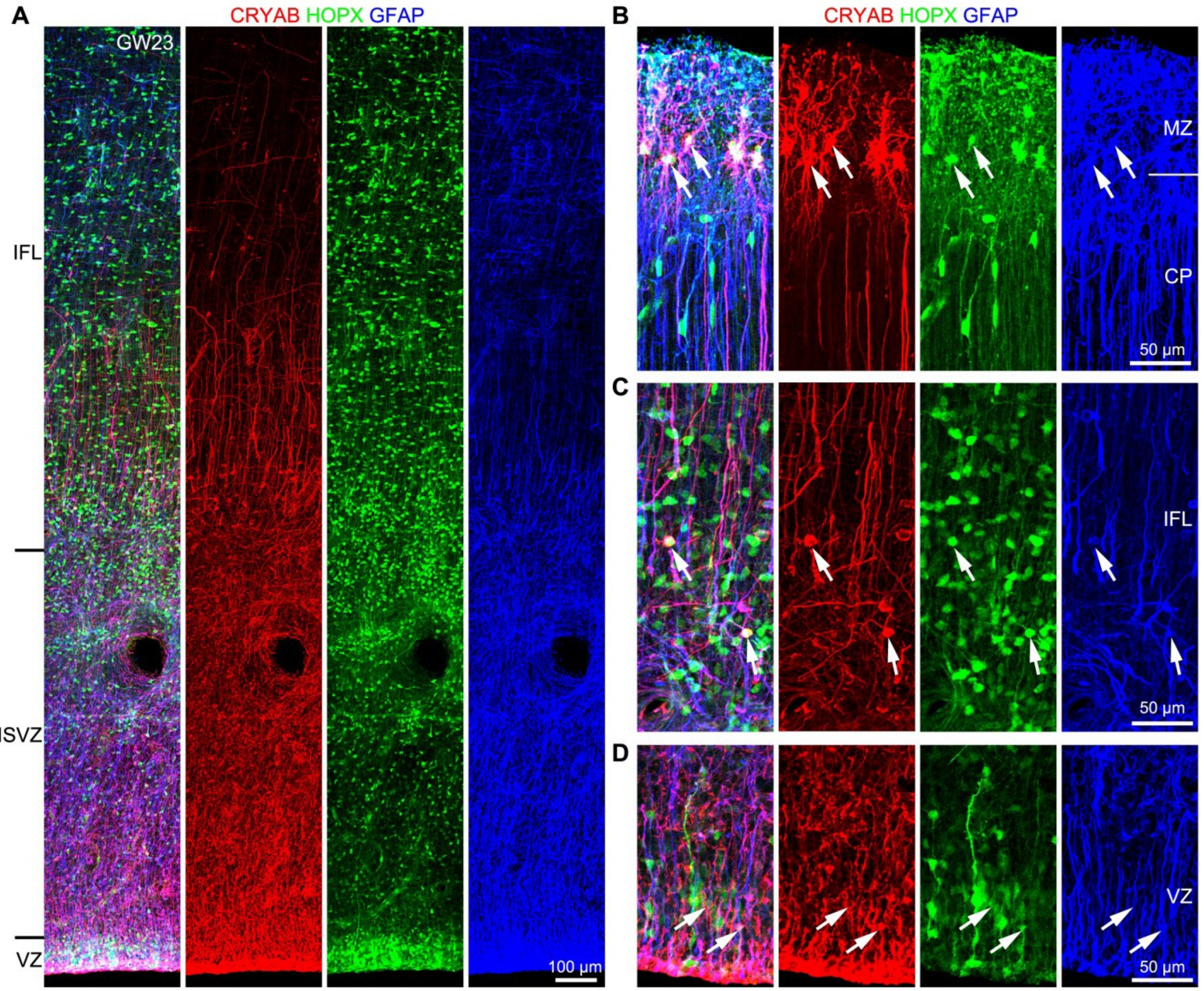

Fig. S3 CRYAB is expressed in tRGs and a few astrocyte lineage cells in the human cortex at GW23. **A** HOPX, CRYAB and GFAP triple immunostained cortical section at GW23. **B** Astrocytes in cortical marginal zone (MZ) expressed CRYAB, HOPX and GFAP (arrows). **C** A few $HOPX^{+}CRYAB^{+}GFAP^{+}$ APCs (arrows) in the cortical IFL. **D** $HOPX^{+}CRYAB^{+}GFAP^{+}$ tRGs (arrows) in the VZ.

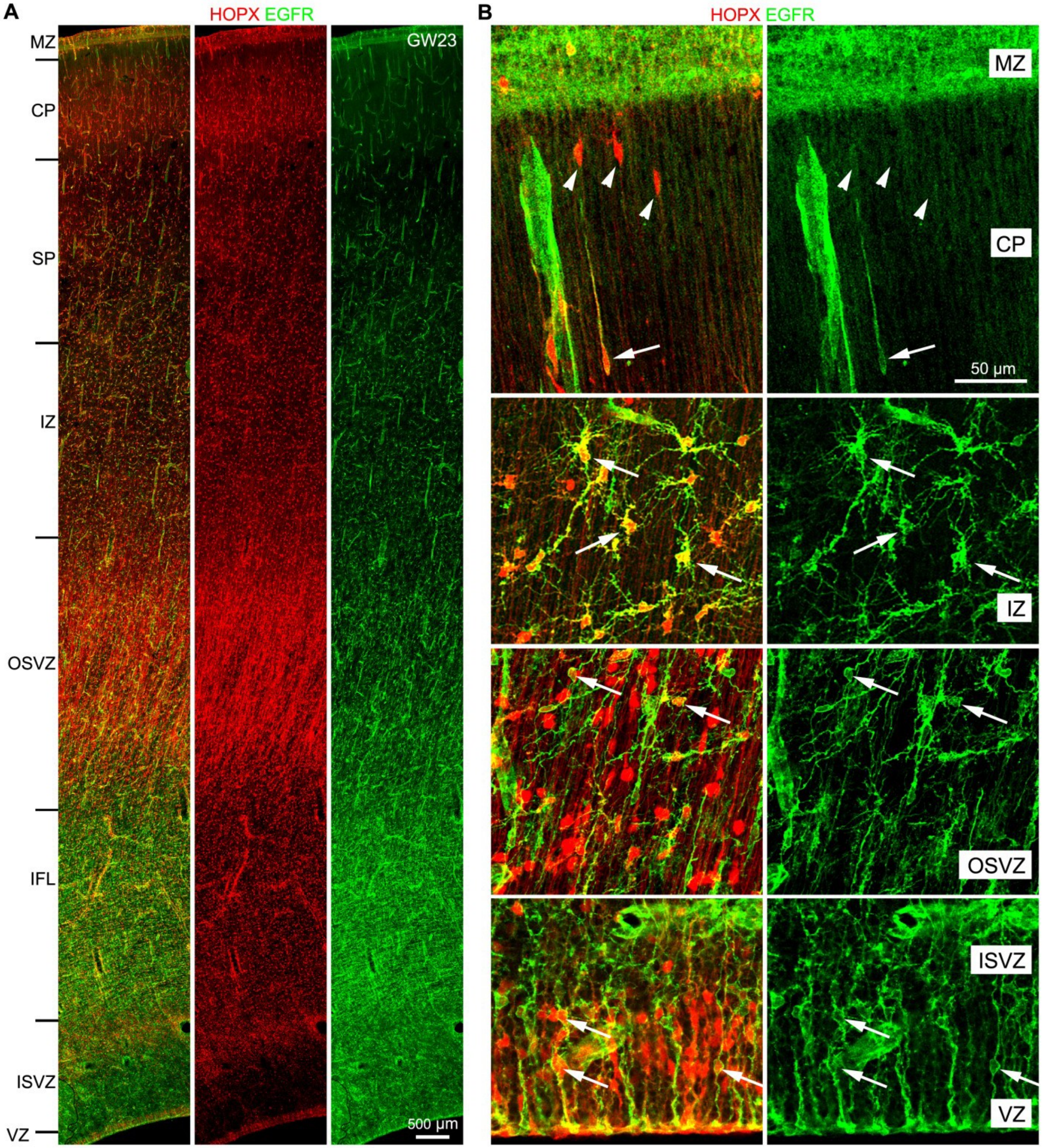

Fig. S4 Human cortical astrocyte lineage cells express HOPX and EGFR. **A** HOPX and EGFR double immunostained GW23 human cortical section. **B** Higher magnification images showing HOPX$^+$EGFR$^+$ APCs (arrows) in the cortical CP, IZ and OSVZ, and HOPX$^+$EGFR$^+$ tRGs (arrows) in the cortical VZ. Note that most of the HOPX$^+$ astrocytes (arrowheads) in the cortical CP downregulated EGFR expression at GW23.

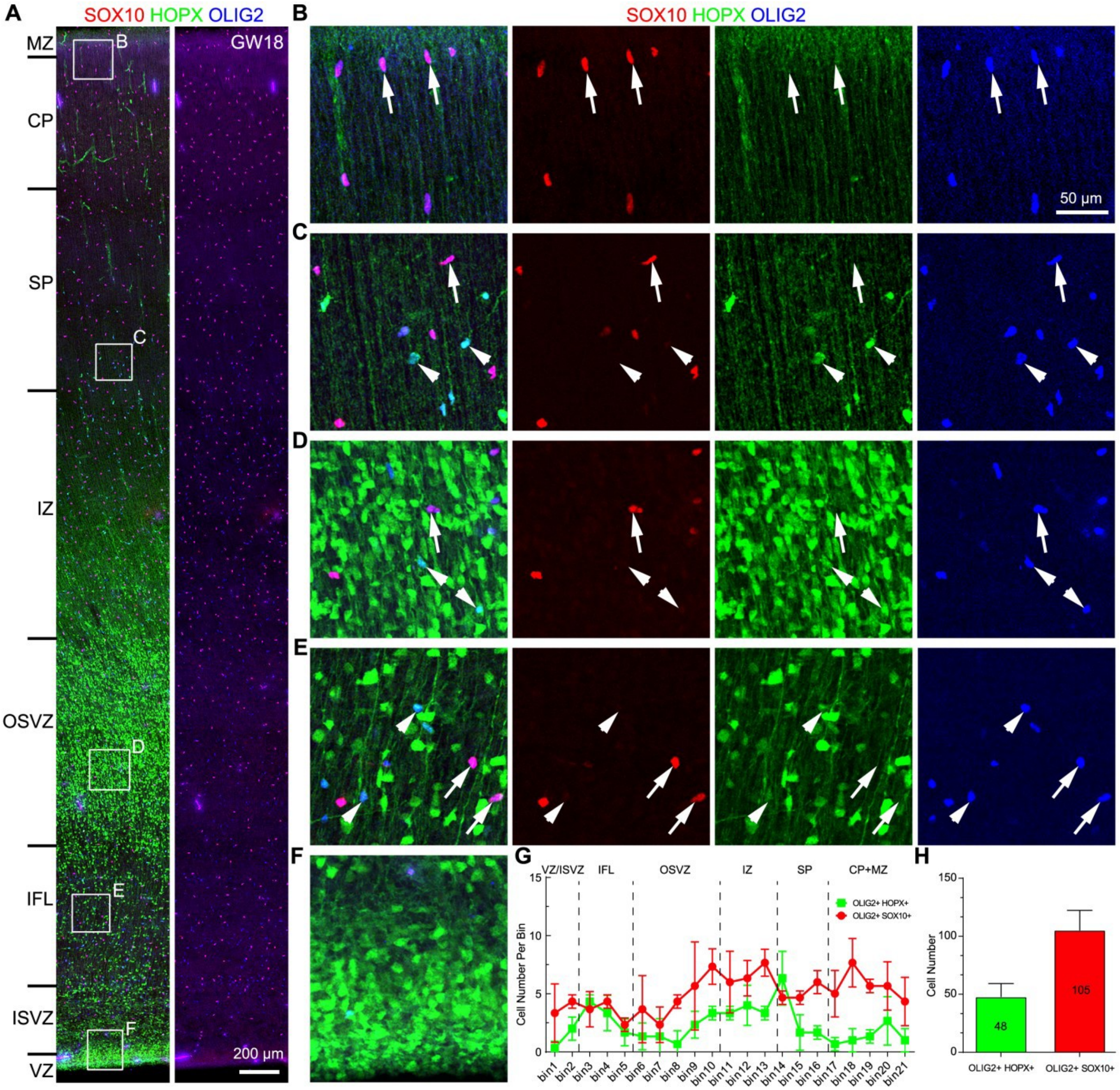

Fig. S5 Human cortical OPCs do not express HOPX. **A** SOX10, HOPX and OLIG2 triple immunostained GW18 human cortical section. **B–F** Higher magnification images of boxed areas in (**A**) showing that all cortical $SOX10^+$ cells (OPCs) expressed OLIG2, but not HOPX (arrows), whereas cortical $HOPX^+$ APCs expressed OLIG2 but not SOX10 (arrowheads). Some $HOPX^+$ cells in the cortical plate expressed extremely low level of OLIG2, indicating that OLIG2 expression is gradually downregulated in immature astrocytes. **G** Numbers of $OLIG2^+SOX10^+$ OPCs and $OLIG2^+HOPX^+$ APCs in the GW18 cortex. **H** Total number of $OLIG2^+HOPX^+$ APCs was about 50% of $OLIG2^+SOX10^+$ OPCs in the GW18 human cortex; most of these OPCs were derived from the MGE, and most of APCs were likely derived from transforming cortical tRGs.

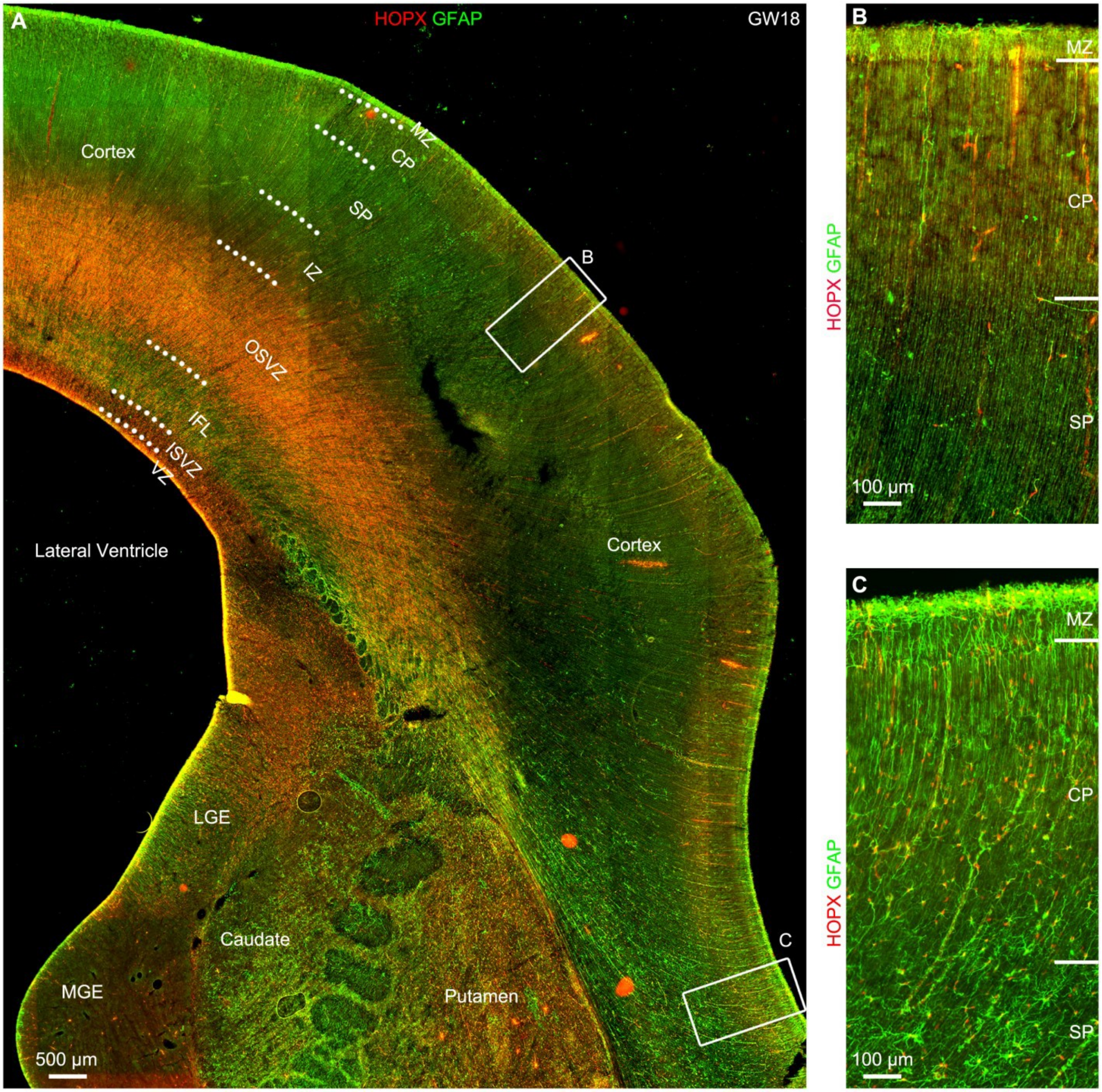

Fig. S6 Human cortical astrocytes are first developed in the ventral cortex. **A** HOPX and GFAP double immunostained GW18 human brain section. **B, C** Higher magnification images of boxed areas in (**A**) showing that there were many more $HOPX^{+}GFAP^{+}$ astrocyte lineage cells in the ventral cortex than that in the dorsal-lateral and dorsal cortex.

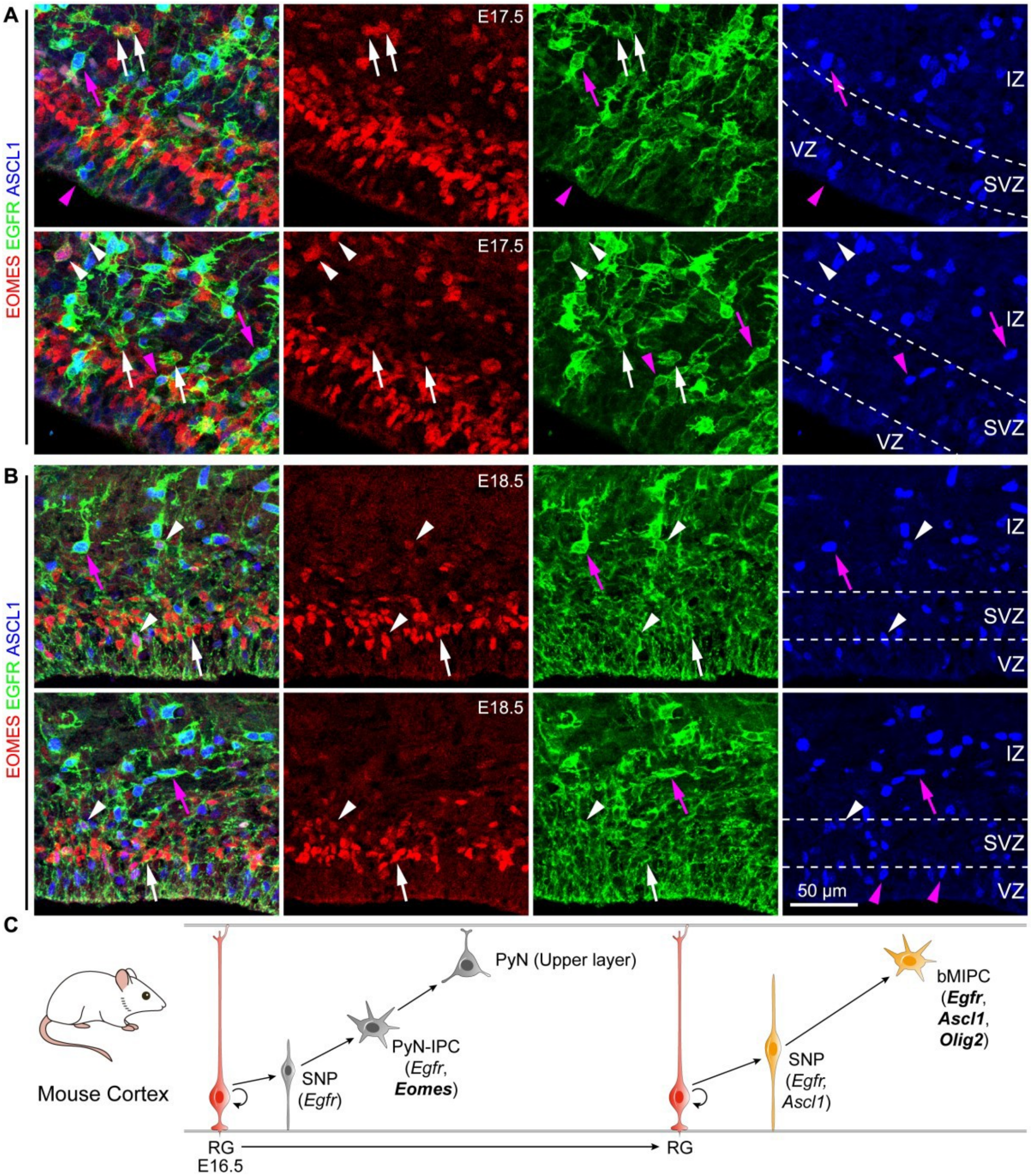

Fig. S7 The direct progeny of cortical EGFR$^+$ apical IPCs in the mouse cortex. **A, B** EGFR, EOMES and ASCL1 triple-immunostained E17.5 and E18.5 mouse cortical sections. White arrows indicated EGFR$^+$EOMES$^+$ PYN-IPCs, magenta arrowheads indicated EGFR$^+$ASCL1$^+$ apical MIPCs (aMIPCs), and magenta arrows indicated EGFR$^+$ASCL1$^+$ bMIPCs. In general, EGFR$^+$ASCL$^+$ apical IPCs were mainly in the cortical VZ and VZ/SVZ border, contacting with the lateral ventricle; EGFR$^+$EOMES$^+$ PyN-IPCs were mainly in the cortical SVZ, whereas EGFR$^+$ASCL1$^+$ bMIPCs were mainly in the cortical SVZ/IZ border and IZ. Note that some EGFR$^+$ASCL1$^+$EOMES$^+$ IPCs (white arrowheads) were also identified. Also note that bMIPCs usually had a large soma (magenta arrows, expressing OLIG2, OLIG1 and MIK67, data not shown), indicating their higher proliferative activity. **C** Summary of progeny of EGFR$^+$ apical IPCs in the mouse cortex. Around E16.5, mouse cortical RGCs begin to generate apical IPCs that express weak EGFR and ASCL1. Initially, these EGFR$^+$ASCL1$^+$ apical IPCs give rise to EGFR$^+$EOMES$^+$ PyN-IPCs that mainly differentiate into upper layer PyNs, but soon, with the accumulation of EGFR and ASCL1 proteins, these EGFR$^+$ASCL1$^+$ aMIPCs give rise to EGFR$^+$ASCL1$^+$OLIG2$^+$ bMIPCs, which in turn generate OPCs, APCs and OBiN-IPCs in the cortex.

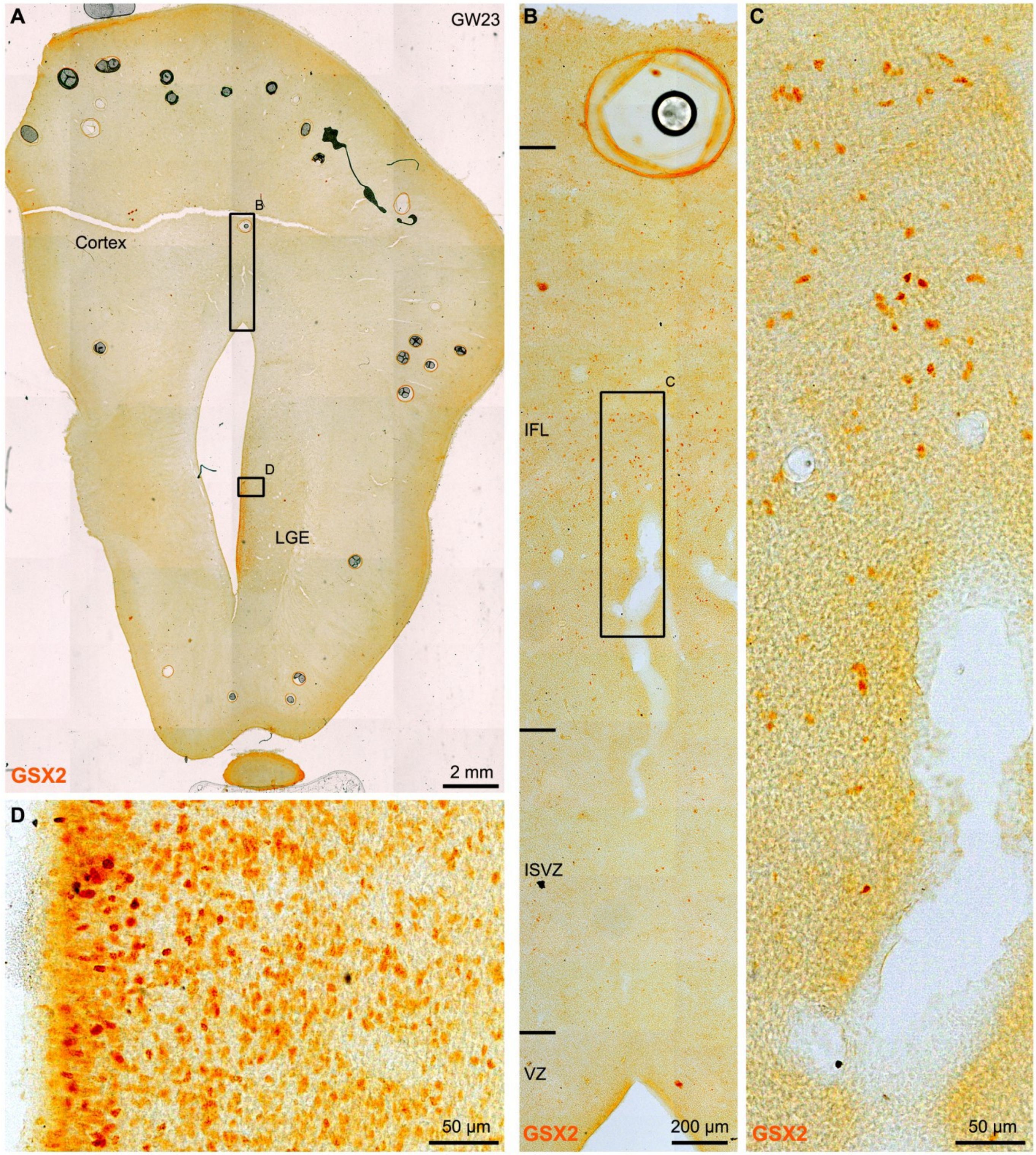

Fig. S8 GSX2 is expressed in the LGE and cortex of the human fetal brain at GW23. **A** Immunohistochemistry for GSX2 on GW23 brain section. **B, C** Higher magnification images showing cortical GSX2^{+} cells in the cortical IFL. **D** Higher magnification image showing the high density of GSX2^{+} cells in the LGE. Note that, in the LGE, GSX2 is expressed in the VZ and SVZ, whereas in the cortex, GSX2 is mainly expressed in the cortical IFL.

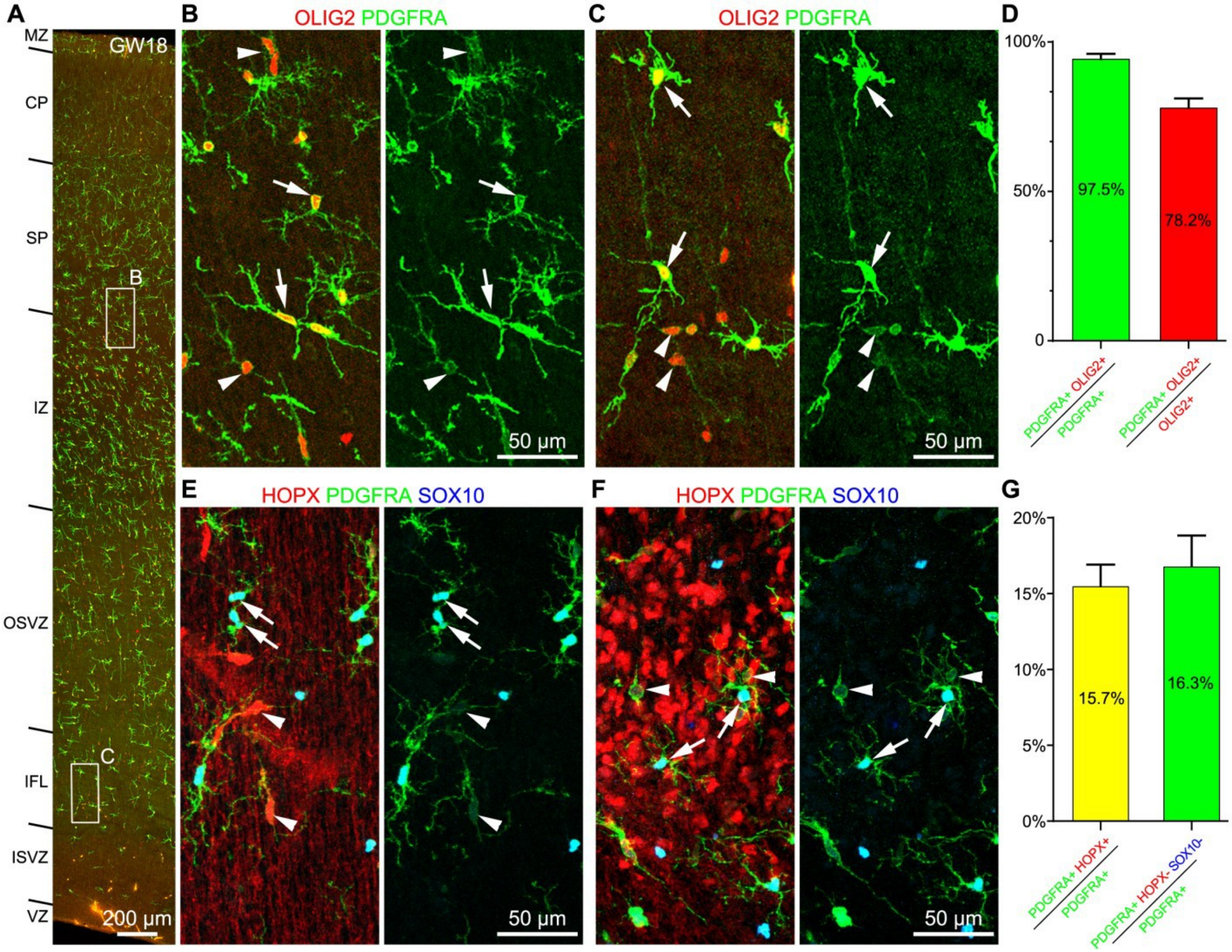

Fig. S9 PDGFRA is expressed in OPCs, bMIPCs and APCs in the human developing cortex. **A** PDGFRA and OLIG2 double immunostained GW18 human cortical section. **B, C** Higher magnification images of boxed areas in (**A**) showing PDGFRA$^+$OLIG2$^+$ cells. Arrows indicated PDGFRA and OLIG2 heavily labeled cells (OPCs). Arrowheads indicated those cells that expressed weak PDGFRA. **D** Quantitative results showed that, in the GW18 cortex, 97.5% of PDGFRA$^+$ cells expressed OLIG2, whereas 78.2% of OLIG2$^+$ cells expressed PDGFRA. **E** PDGFRA was weakly expressed in some cortical HOPX$^+$ APCs (arrowheads). **F** PDGFRA was weakly expressed in some cortical bMIPCs (arrowheads); these cells expressed neither HOPX nor SOX10, consistent with scRNA-Seq analysis. Arrows in (**E** and **F**) indicated PDGFRA$^+$SOX10$^+$ OPCs. **G** From the cortical IFL to SP, 15.7% of PDGFRA$^+$ cells expressed HOPX, suggesting that they are APCs. In the cortical IFL and OSVZ, 16.3% of PDGFRA$^+$ cells expressed neither HOPX nor SOX10, suggesting that they are bMIPCs.

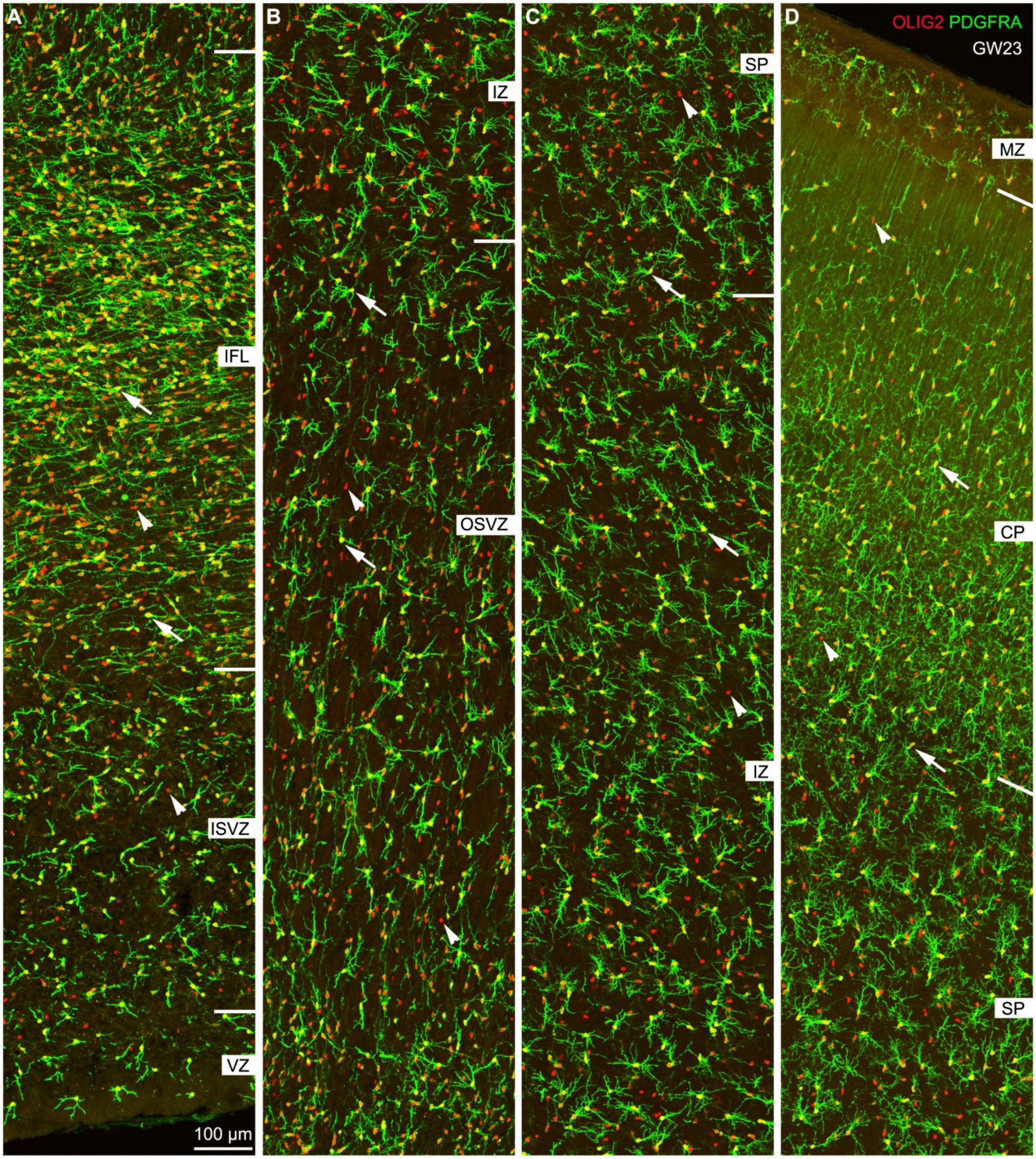

Fig. S10 A large number of OPCs are in the human cortex at GW23. **A–D** Heavily PDGFRA⁺OLIG2⁺ OPCs in the GW23 human cortex were shown. Note that almost all cortical PDGFRA⁺ OPCs (arrows) expressed OLIG2, but there were some OLIG2⁺ cells (arrowheads) that did not express PDGFRA. These OLIG2⁺ but PDGFRA immunonegative cells could be cortical bMIPCs or APCs.

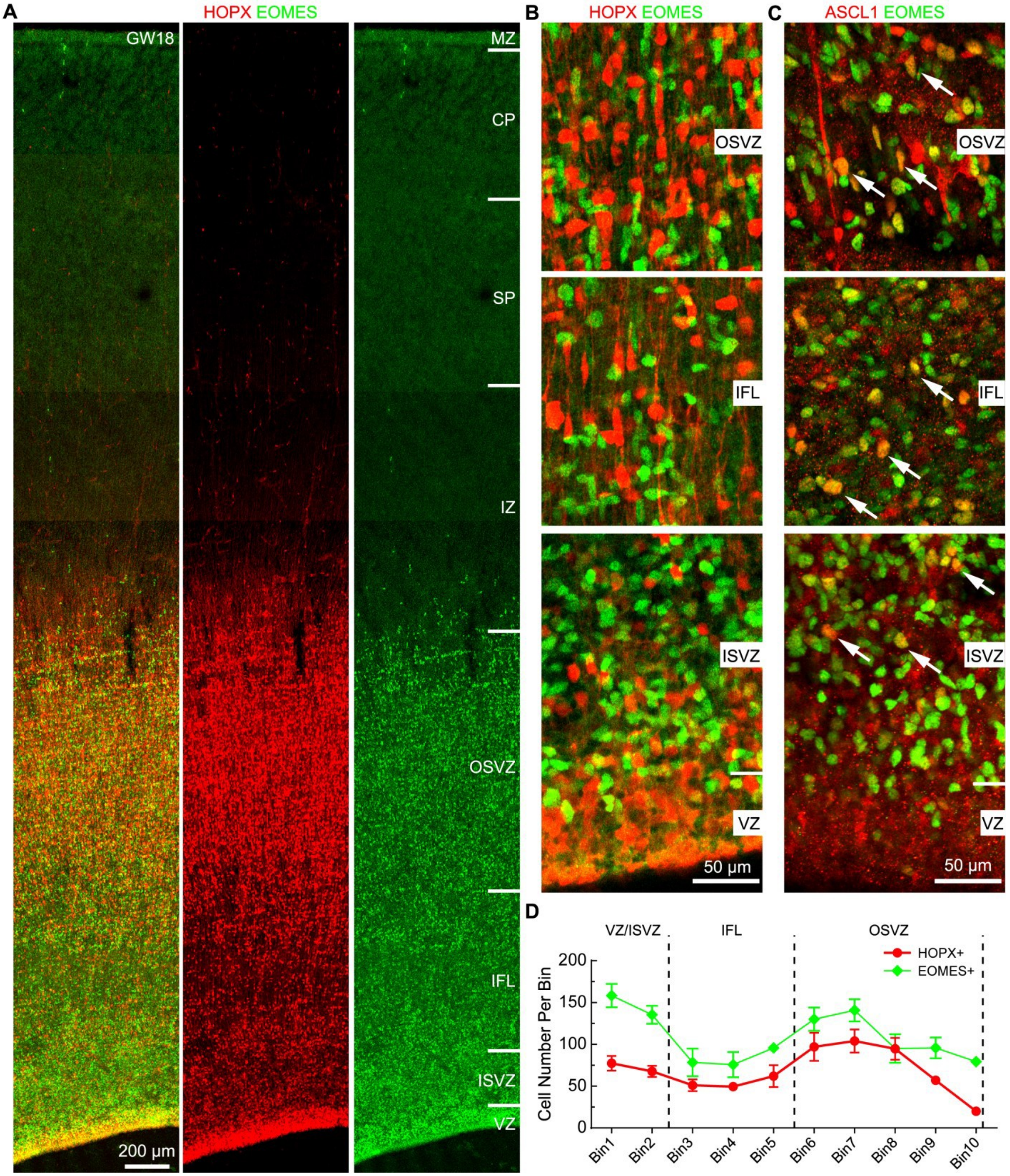

Fig. S11 There are two germinal zones for the PyN genesis in the human cortex at GW18. **A** Double immunohistochemistry for HOPX and EOMES. **B** Higher magnification images showing HOPX$^+$ tRGs in the VZ and HOPX$^+$ oRGs in the IFL and OSVZ. HOPX$^+$ tRGs and HOPX$^+$ oRGs did not express EOMES. **C** Many tRG-derived and oRG-derived EOMES$^+$ PyN-IPCs expressed ASCL1. **D** Numbers of HOPX$^+$ cells and EOMES$^+$ PyN-IPCs in the GW18 cortical VZ, ISVZ, IFL and OSVZ.

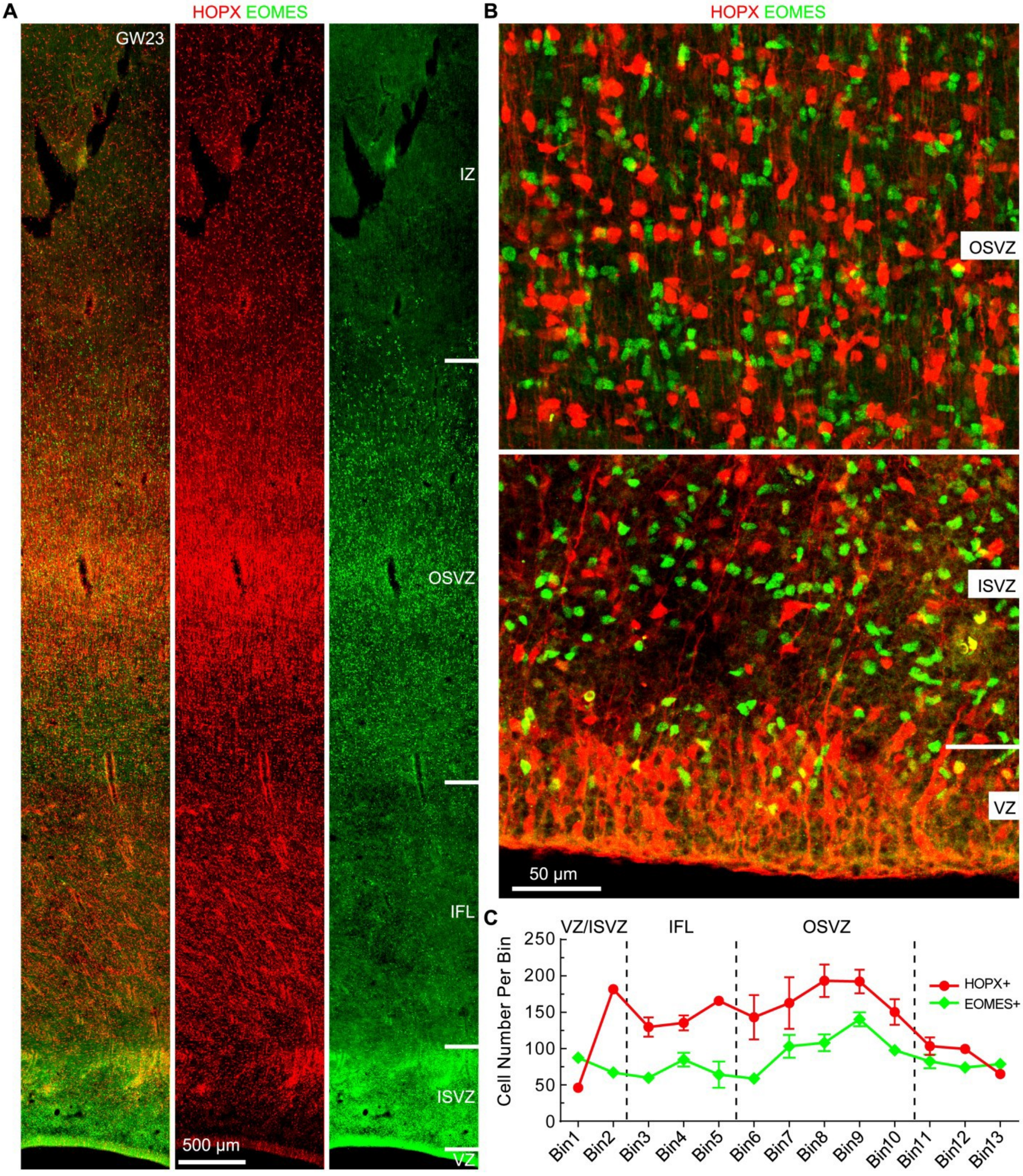

Fig. S12 PyN genesis in the cortex of the human fetal brain at GW23. **A** HOPX and EOMES double immunostained GW23 human cortical section. **B** Higher magnification images showing HOPX$^+$ tRGs and HOPX$^+$ oRGs, and their progeny: EOMES$^+$ PyN-IPCs. HOPX$^+$ cells did not express EOMES. **C** Quantification of numbers of HOPX$^+$ cells and EOMES$^+$ cells in the GW23 cortical VZ, ISVZ, IFL and OSVZ.

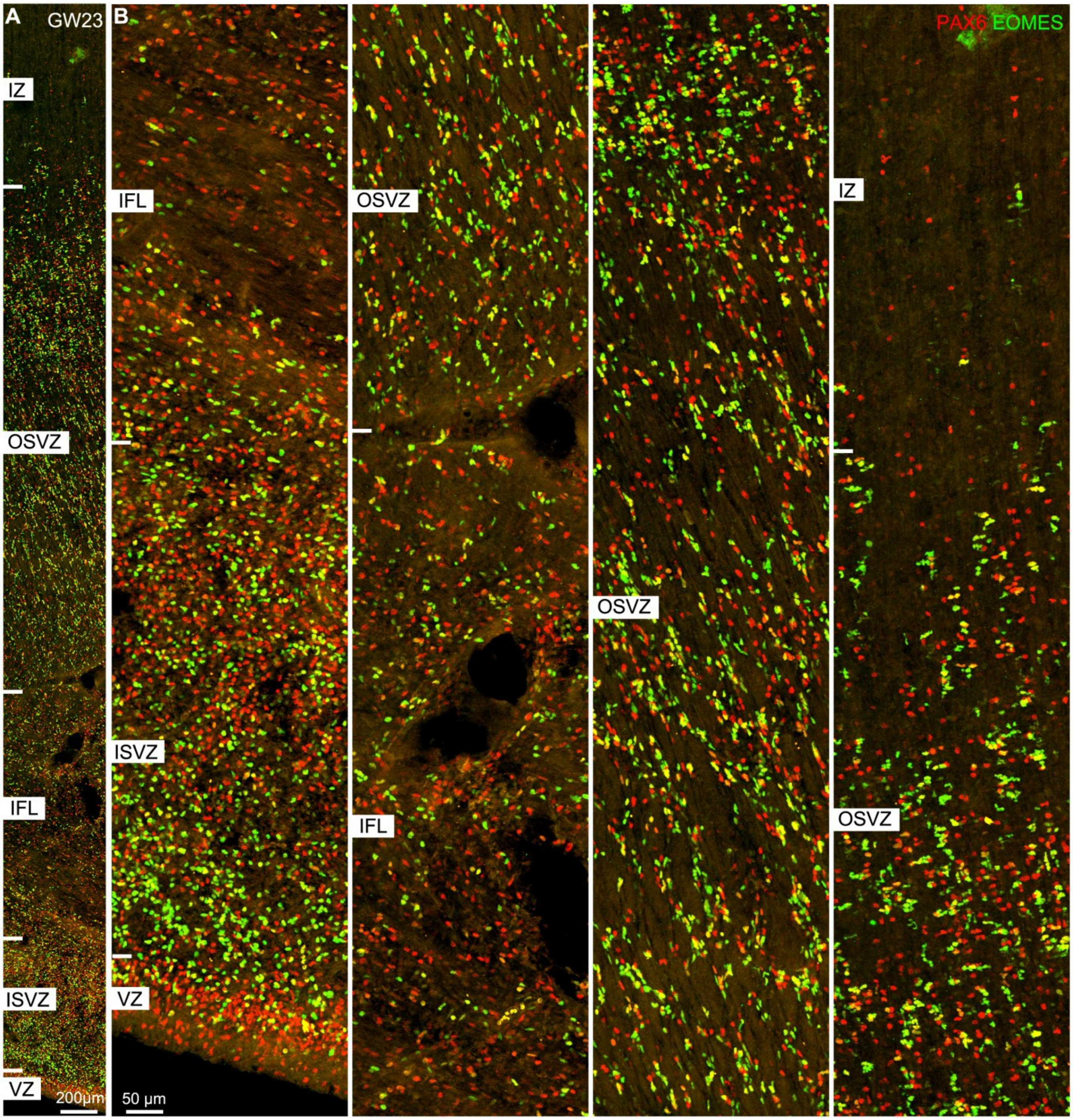

Fig. S13 PAX6 & EOMES double immunohistochemistry reveals PyN genesis in the GW23 human cortex. **A** PAX6 and EOMES double immunostained GW23 human cortical section. **B** Higher magnification images showing that virtually all EOMES$^+$ PyN-IPCs expressed PAX6.
